# Extinction of Hepatitis C Virus by Ribavirin in Hepatoma Cells Involves Lethal Mutagenesis

**DOI:** 10.1371/journal.pone.0071039

**Published:** 2013-08-16

**Authors:** Ana M. Ortega-Prieto, Julie Sheldon, Ana Grande-Pérez, Héctor Tejero, Josep Gregori, Josep Quer, Juan I. Esteban, Esteban Domingo, Celia Perales

**Affiliations:** 1 Centro de Biología Molecular “Severo Ochoa” (CSIC-UAM), Consejo Superior de Investigaciones Científicas (CSIC), Campus de Cantoblanco, Madrid, Spain; 2 Instituto de Hortofruticultura Subtropical y Mediterránea “La Mayora” (IHSM-UMA-CSIC), Departamento de Biología Celular, Genética y Fisiología, Universidad de Málaga, Campus Teatinos, Málaga, Spain; 3 Departamento de Bioquímica y Biología Molecular I, Universidad Complutense de Madrid, Madrid, Spain; 4 Centro de Investigación Biomédica en Red de Enfermedades Hepáticas y Digestivas (CIBERehd), Barcelona, Spain; 5 Liver Unit, Internal Medicine Lab. Malalties Hepàtiques, Vall d'Hebron Institut Recerca-Hospital (VHIR-HUVH), Barcelona, Spain; 6 Universitat Autónoma de Barcelona, Barcelona, Spain; 7 Roche Diagnostics, S.L., Sant Cugat del Vallès, Spain; Institut Pasteur, France

## Abstract

Lethal mutagenesis, or virus extinction produced by enhanced mutation rates, is under investigation as an antiviral strategy that aims at counteracting the adaptive capacity of viral quasispecies, and avoiding selection of antiviral-escape mutants. To explore lethal mutagenesis of hepatitis C virus (HCV), it is important to establish whether ribavirin, the purine nucleoside analogue used in anti-HCV therapy, acts as a mutagenic agent during virus replication in cell culture. Here we report the effect of ribavirin during serial passages of HCV in human hepatoma Huh-7.5 cells, regarding viral progeny production and complexity of mutant spectra. Ribavirin produced an increase of mutant spectrum complexity and of the transition types associated with ribavirin mutagenesis, resulting in HCV extinction. Ribavirin-mediated depletion of intracellular GTP was not the major contributory factor to mutagenesis since mycophenolic acid evoked a similar decrease in GTP without an increase in mutant spectrum complexity. The intracellular concentration of the other nucleoside-triphosphates was elevated as a result of ribavirin treatment. Mycophenolic acid extinguished HCV without an intervening mutagenic activity. Ribavirin-mediated, but not mycophenolic acid-mediated, extinction of HCV occurred via a decrease of specific infectivity, a feature typical of lethal mutagenesis. We discuss some possibilities to explain disparate results on ribavirin mutagenesis of HCV.

## Introduction

Hepatitis C virus (HCV) infections affect about 180 million people worldwide, and an estimated 75% of newly infected patients progress towards a chronic infection, which constitutes a risk for severe liver diseases such as cirrhosis and hepatocarcinoma [1]–[4]. HCV is an hepacivirus of the *Flaviviridae* family that displays the error-prone replication and quasispecies dynamics typical of RNA viruses [3], [5]–[7]. No vaccine is available to prevent HCV infections or disease, and the current standard of care (SOC) treatment consists of the combination of pegylated interferon-α (IFN-α) and the purine nucleoside analogue ribavirin (1-*β*-D-ribofuranosyl-1-*H*-1,2,4-triazole-3-carboxamide) (Rib) (IFN-α+Rib). However, on average only about 60% of the chronically infected patients show a sustained virological response that results in virus clearance [8]–[11]. The mechanism of anti-HCV activity of IFN-α+Rib and the reasons for treatment failure are largely unknown. HCV evolving in hepatoma cells in culture can find multiple mutation pathways to acquire resistance to IFN-α, and resistant HCV populations display decreased sensitivity to a IFN-α+Rib combination treatment [12]. New therapeutic options have been opened with the development of directly acting antiviral agents (DAAs), but it is not clear which new combinations will be the most effective, although inclusion of Rib appears to be important [13]–[17].

The prior experience gained with the treatment of infections by highly variable RNA viruses that exhibit quasispecies dynamics, in particular human immunodeficiency virus type 1 (HIV-1), anticipates that HCV variants with different degrees of resistance to one or multiple anti-HCV inhibitors will be selected with high probability in the course of the new treatments [18]–[25]. Rapid selection of antiviral-resistant mutants is a direct consequence of quasispecies dynamics and one of the main reasons that impulsed research on a new antiviral strategy termed virus entry into error catastrophe or lethal mutagenesis. It is based on one of the corollaries of quasispecies theory that asserts that a sufficient increase of error rate during genome replication should result in the loss of the information conveyed by the replicative system [26], [27]. In agreement with these and other theoretical treatments [28]–[34], virus extinction associated with enhanced mutagenesis has been documented with several RNA viruses in cell culture and *in vivo* [35]–[54]. Rib is one of the mutagenic agents that are currently used in the investigations on lethal mutagenesis because it has been licensed for human use for several decades, and proven to be mutagenic first for poliovirus (PV) [37], [55], [56], and then for several other RNA viruses [35], [40], [48], [49], [51], [57]–[62], but not for others [63]–[65]. It is not clear whether Rib exerts its anti-HCV activity through mutagenesis or other mechanisms [58], [66]–[77]. Alternative antiviral mechanisms of Rib are: (i) immunomodulation and enhancement of the Th1 antiviral immune response [78], [79]; (ii) up-regulation of genes involved in IFN signaling [80], [81]; (iii) inhibition of viral RNA-dependent RNA polymerases (RdRps) [82]–[87]; (iv) depletion of intracellular GTP levels [88]; and (v) inhibition of mRNA cap formation [89].

While some studies in cell culture have suggested a mutagenic activity of Rib on HCV [58], [73], [76], [90]–[92], other investigations have failed to provide evidence of Rib mutagenesis [93], [94]. Elucidation of the anti-HCV mechanism of Rib is highly relevant because it may influence the design of antiviral protocols. Recent model studies with different virus-host systems have indicated that when a mutagenic agent participates in therapy, a sequential administration of an inhibitor first, followed by a mutagenic agent may be more effective than the corresponding combination therapy to achieve virus extinction [49], [95]–[97]. The advantage of a sequential administration depends on the concentrations of mutagen and inhibitor used, and it requires that one of the drugs used for therapy acts as a mutagenic agent [95]–[97]. Thus, with the advent of DAAs, it is important to clarify the mechanism of Rib activity against HCV to compare the effectiveness of alternative antiviral protocols first in cell culture and then in animal models and clinical trials.

**Figure 1:**
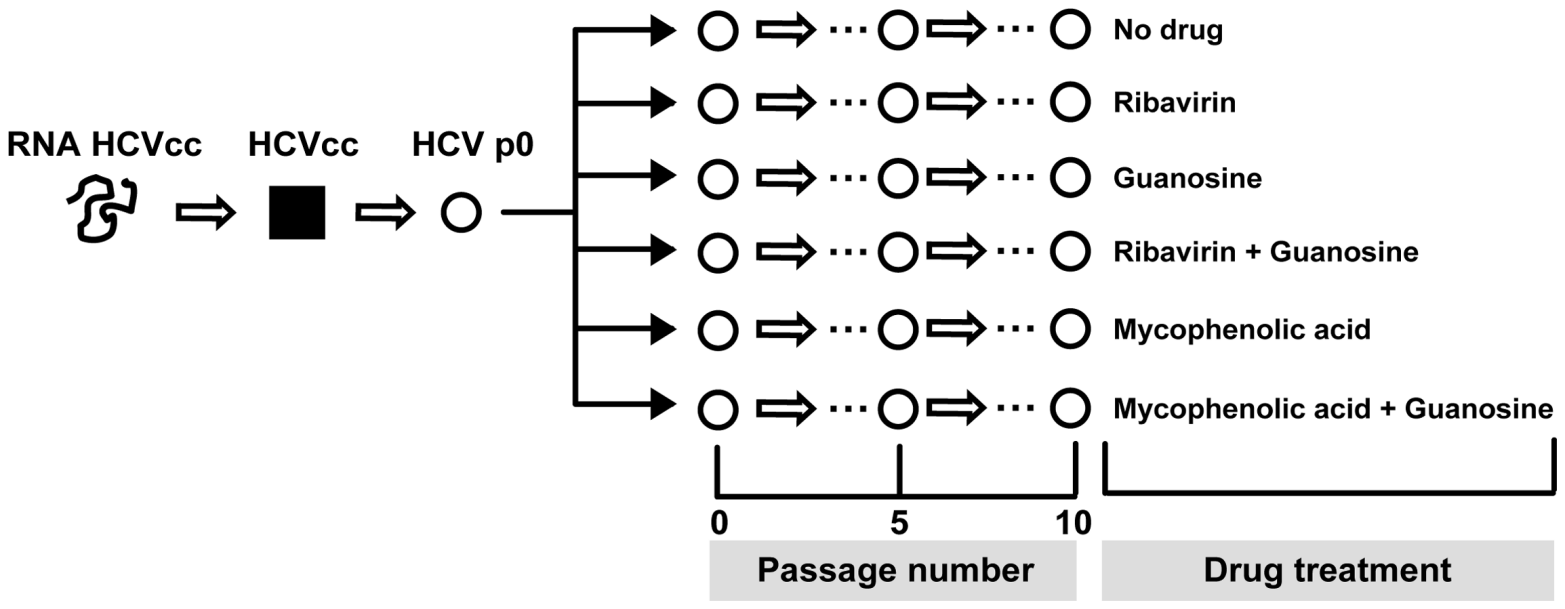
Scheme of serial hepatitis C virus passages performed in the present study. The initial clonal population HCVcc (filled square) was prepared by transcription of plasmid Jc1 FLAG (p7-nsGluc2A) (RNA HCVcc) and electroporation into Lunet cells, as described in Materials and Methods. The amplified virus HCVp0 was subjected to serial passages in the presence of different drugs or drug combinations, as indicated; uncloned populations are depicted as empty circles. Infections with the replication-negative HCV mutant GNN were carried out in parallel (not shown in this scheme). The multiplicity of infection (MOI), drug concentrations, and the various analyses performed with different HCV populations are detailed in the corresponding experiments.

The development of systems which allow infection of cultured cells with HCV particles containing the entire viral genome [98]–[100] permits addressing HCV evolution over extended periods of time (serial infections) in the presence of antiviral agents, without possible confounding effects derived from host cell evolution [12]. Here we study the effect of exposure of HCV in cell culture [12], [101] to several drugs or drug combinations in a multiple passage design, as depicted in Fig. 1. The results indicate that Rib is mutagenic for HCVp0, and that Rib-mediated, but not mycophenolic acid-mediated, HCV extinction displays the attributes of lethal mutagenesis.

## Results

### Cytotoxicity and inhibitory activity of ribavirin and mycophenolic acid

**Figure 2:**
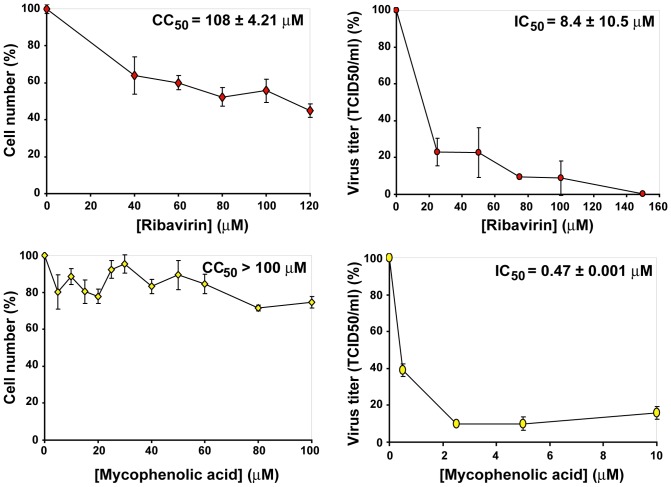
Quantification of cytotoxicity for Huh-7.5 cells and inhibition of HCV infectious progeny production by ribavirin and mycophenolic acid. Determinations of cytotoxic concentration 50 (CC_50_) (left panels) and the drug concentration required for 50% inhibition, or inhibitory concentration 50 (IC_50_) (right panels) were carried out in triplicate. Values and standard deviations were calculated using the program Sigma Plot. Experimental conditions for cell growth, HCV infection, determination of cell viability and HCV infectivity are described in Materials and Methods.

To determine cytotoxicity of the drugs (concentration that reduces cell viability by 50%, CC_50_) used in the present study, Huh-7.5 reporter cells were incubated with increasing concentrations of Rib or mycophenolic acid and cell viability determined after 72 h of treatment. To quantify the inhibition of infectious HCV progeny production (drug concentration that produces a 50% decrease in progeny production, IC_50_), Huh-7.5 cells were infected with HCVp0 at a multiplicity of infection (MOI) of 0.2–0.5 TCID_50_/cell in the presence of increasing concentrations of the drugs and infectious progeny production measured. From the data, CC_50_ and IC_50_ values were calculated (Fig. 2), that yielded a therapeutic index (TI) (CC_50_/IC_50_) of 12.8, and >212.8 for Rib and mycophenolic acid, respectively. These values served as guide for the choice of drug concentrations in the experiments.

### Influence of infection conditions on the response of HCV to ribavirin treatment during replication in human hepatoma cells

**Figure 3:**
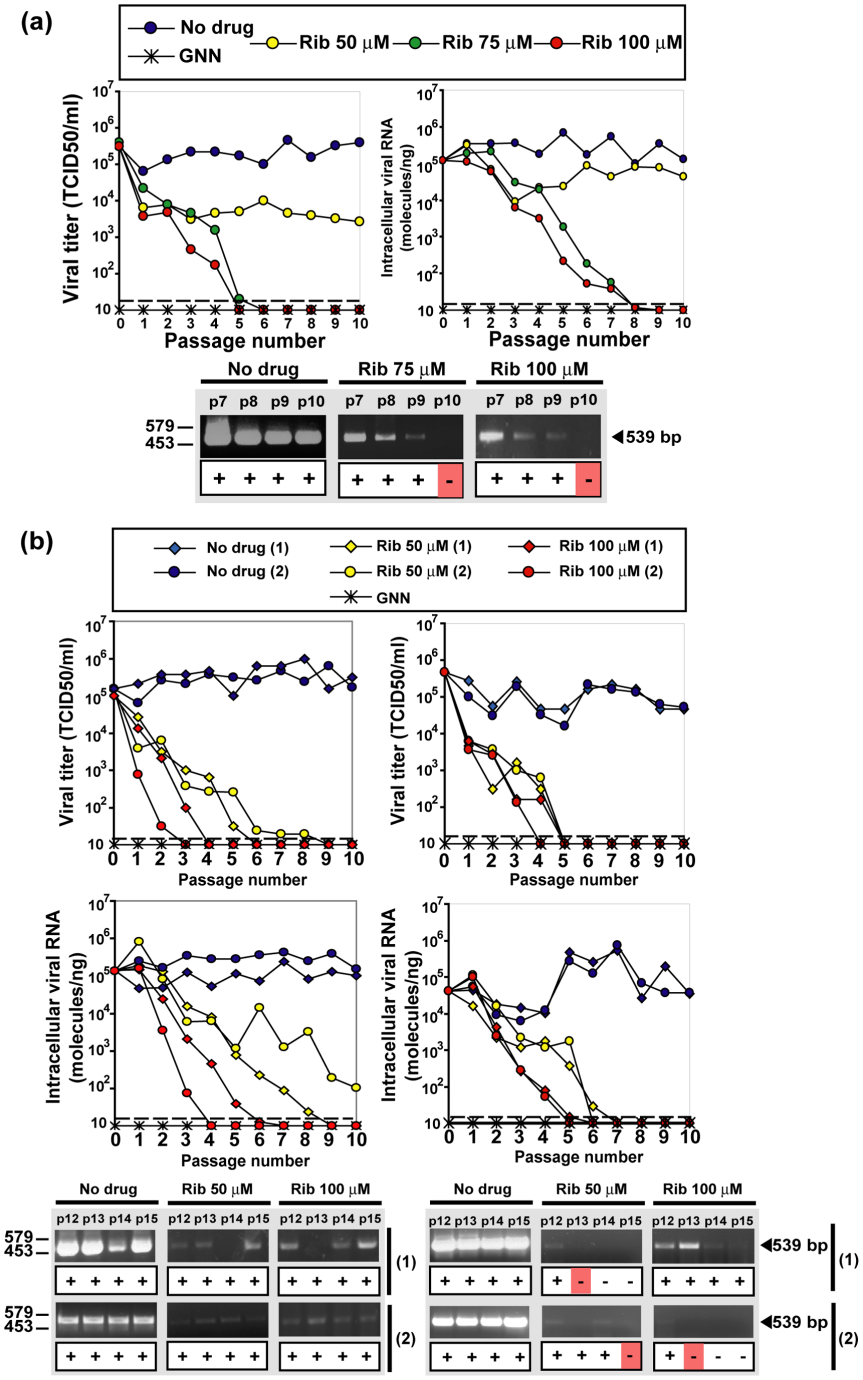
The effect of ribavirin on infectious HCV progeny production and intracellular HCV RNA. (a) Huh-7.5 reporter cells were infected with HCVp0 at a MOI of 1–2 TCID_50_/cell (10^5^ Huh-7.5 reporter cells infected with 1×10^5^ to 2×10^5^ TCID_50_ of HCVp0), in the absence or presence of the Rib concentrations indicated in the upper box. Infections with HCV GNN were carried out in parallel (negative control). The progeny from each infection was used to infect fresh cells, as described in Materials and Methods. Virus infectivity was determined in the cell culture supernatant (left panel), and intracellular viral RNA measured by quantitative RT-PCR (right panel). The discontinuous line parallel to the abscissa indicates the limit of detection of infectivity and viral RNA. Below, RT-PCR amplification bands using a highly sensitive HCV-specific amplification protocol that yields a 539 bp fragment, using as template total intracellular RNA from the infection series and passage numbers indicated at the top of the corresponding lanes; +, −: presence or absence of amplification band; the position of DNA size markers is indicated on the left. (b) Same as in (a) but with infections carried out at an initial MOI of 0.1 – 0.2 TCID_50_/cell, using two different protocols: 4×10^6^ Huh-7.5 reporter cells infected with 4×10^5^ to 8×10^5^ TCID_50_ of HCVp0 (duplicate assays, named 1 and 2, left panels), and 4×10^5^ Huh-7.5 reporter cells infected with 4×10^4^ to 8×10^4^ TCID_50_ of HCVp0 (duplicate assays, named 1 and 2, right panels). Symbols and RT-PCR amplification bands shown at the bottom were obtained as is explained in part (a). Procedures are described in Materials and Methods.

The parental HCVp0 virus [12], [101] was prepared as described in Materials and Methods, and used to investigate the response of HCV to Rib. HCVp0 was subjected to 10 serial passages in Huh-7.5 cells using either high (1–2 TCID_50_/cell) or low (0.1–0.2 TCID_50_/cell) initial MOI, in the absence or presence of different Rib concentrations. The results show a consistent decay of progeny infectivity and of intracellular HCV RNA as a result of Rib treatment, while a sustained viral replication was observed in the absence of Rib (Fig. 3). An exception was the decreased but continued replication of HCV in the presence of 50 μM Rib in the infection at high MOI (Fig. 3a). In all other cases, infectivity and viral RNA decreased to levels below the corresponding limits of detection by passages 3 to 9 (Fig. 3a, b). Decay of infectivity in the cell culture supernatant always preceded decay of intracellular viral RNA, an observation previously made during mutagenesis-based extinction of lymphocytic choriomeningitis virus (LCMV) [42].

In previous studies on lethal mutagenesis of foot-and-mouth disease virus (FMDV) and LCMV we defined a double criterion to consider the virus extinct: (i) absence of infectivity following three blind passages of the undiluted cell culture supernatant in the host cells in the absence of any drug, and (ii) absence of intracellular viral RNA using a highly sensitive RT-PCR amplification protocol [47], [52], [57]. When these criteria were applied to the Rib-treated HCV populations, no infectivity could be rescued when supernatants devoid of detectable infectivity were further passaged in the absence of Rib. However, some residual HCV RNA persisted as judged by an amplification band (above the background level of the negative control) even when the treatment was extended to 15 passages (Fig. 3b).

### Mutant spectrum complexity in hepatitis C virus populations passaged in the absence or presence of ribavirin

**Figure 4:**
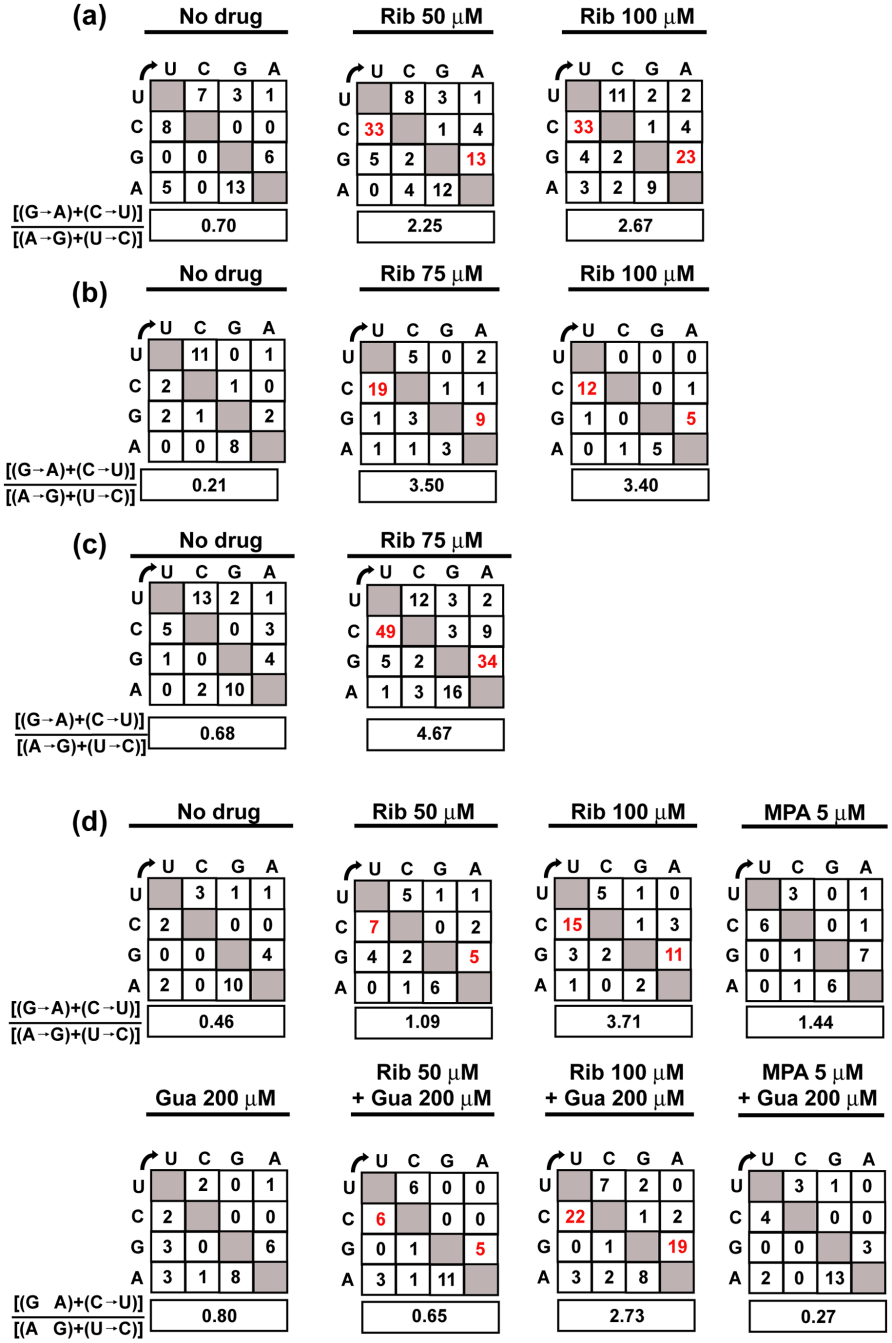
Matrix of nucleotide substitutions in the mutant spectrum of hepatitis C virus populations passaged in the absence or presence of drugs or drug combinations. Mutation types (from nucleotides written vertically to those written horizontally) are counted for the individual components of the mutant spectrum relative to the corresponding consensus sequence of the population. Drug concentrations present during HCV passages are written at the top of each matrix (abbreviated as: ribavirin, Rib; mycophenolic acid, MPA; guanosine, Gua). When Rib was present, the types of mutations expected from Rib mutagenesis are highlighted in red, and their ratio to the other transition types is boxed below each matrix. (a) HCV populations subjected to three passages at an initial MOI of 0.1–0.2 TCID50/cell; the populations are those described in Fig. 3b and Tables 1, S1, S2 and S3. (b) HCV populations subjected to four or five passages at an initial MOI of 1–2 TCID50/cell; the populations are those described in Fig. 3a and Tables 2 and S4. (c) Same as (b) but for HCV populations at passage 4 analyzed by UDPS, including mutations that are repeated in different amplicons; the populations are those described in Tables 3 and 5. (d) HCV populations subjected to three passages at a MOI of 0.1–0.2 TCID50/cell in the presence of Rib or MPA with or without Gua; the populations are those described in Figs. 5 and 6, and Tables 4, 5, S6 and S7. Procedures are described in Materials and Methods.

To examine whether the decrease of infectivity upon HCV replication in the presence of Rib was accompanied with a mutagenic activity of Rib, the mutant spectrum complexity at the E2-, NS5A- and NS5B-coding regions (chosen because they encode structural and non-structural proteins displaying different degrees of conservation) of several HCV populations was evaluated (Table 1). NS5A and NS5B of populations passaged three times at low MOI in the presence of Rib underwent a significant increase of mutation frequency (1.4- to 3.0-fold with 50 μM Rib, and 1.6- to 4.8-fold with 100 μM Rib), relative to the population passaged in absence of Rib (p<0.05 to p<0.0005; χ^2^ test). Statistically significant increases of nucleotide diversity were also observed for increasing Rib concentrations (NS5A p<0.004; NS5B p<0.04 and p<0.005; Permutation test) (Table 1). Ribavirin induces preferentially G→A and C→U transitions in HCV and other RNA viruses [35], [56], [57], [77], [102]. While the percentage of transitions relative to the total number of mutations did not vary as a result of Rib treatment (79.1%, 75.6% and 79.4% in the absence and presence of 50 and 100 μM Rib, respectively), the corresponding percentage of [(G→A)+(C→U)] transitions increased from 32.5% in the absence of Rib to 51.7% and 57.7% in the presence of 50 and 100 μM Rib, respectively, reflected also in an increase of the [(G→A)+(C→U)]/[(A→G)]+[(U→C)] ratio (Fig. 4a). The mutational bias reinforces the conclusion that Rib acts as a HCV mutagen. [The amino acid substitutions and their score according to the PAM 250 substitution matrix [103] – a measure of their probability of occurrence – are listed in Tables S1, S2 and S3.

**Table 1:** Quasispecies analysis of HCVp0 populations passaged in the absence or presence of ribavirin^a^.

Genomic region^b^	Ribavirin conc. (µM)^a^	Number of nt analyzed (clones/haplotypes)^c^	Mutation frequency^d^	Nucleotide diversity e
				π. 10^3^ (95% CI)
	**0**	15,122 (15/9)	9.3×10^−4^	1.11 (0.73–1.58)
**E2 (nt 1490–2590)**	**50**	18,860 (17/14)	1.3×10^−3^	1.49 (1.15–2.01)
	**100**	20,657 (21/16)	1.5×10^−3^	1.74 (1.33–2.51)
	**0**	29,704 (22/18)	7.7×10^−4^	1.16 (0.93–1.51)
**NS5A (nt 6269–7666)**	**50**	23,766 (17/17)	1.4×10^−3^	1.54 (1.16–2.11)
	**100**	21,396 (20/20)	2.1×10^−3^	2.43 (1.99–3.44)
	**0**	22,691 (18, 10/5, 3)	2.5×10^−4^	0.74 (0.53–1.03)/0.23 (0.11–0.56)
**NS5B (nt 7667**–**8442/ 8443**–**9442)**	**50**	37,862 (24, 22/17, 9)	7.6×10^−4^	1.60 (1.22–2.10)/0.50 (0.26–0.87)
	**100**	16,676 (12, 11/7, 8)	1.2×10^−3^	1.25 (0.79–1.79)/1.39 (0.74–2.30)

a The population analyzed correspond to passage 3 of the infections at an initial MOI of 0.1 to 0.2 TCID_50_/cell described in Fig. 3b.

b The HCV genome residue numbering corresponds to the JFH-1 genome (accession number #AB047639). The NS5B-coding region was covered by two overlapping amplifications.

c The parenthesis indicates the number of clones analyzed, followed by the number of haplotypes (number of different RNA sequences); some clones did not contain the full length sequence; when the alignment of the sequenced region was correct such clones were entered in the calculation.

d Average number of mutations per nucleotide relative to the corresponding consensus sequence. Mutation types are summarized in Fig. 4a and their position in the HCV genome and deduced amino acid substitutions are given in Tables S1, S2 and S3.

e The calculation of nucleotide diversity (π) and 95% confidence interval (CI) is explained in Materials and Methods.

To further investigate the mutagenic activity of Rib, mutant spectrum complexity of the NS5A-coding region in the populations passaged at a MOI of 1 to 2 TCID_50_/cell was investigated by molecular cloning and Sanger sequencing, and ultra-deep pyrosequencing. The analysis of molecular clones (Table 2) indicates a significant increase of mutation frequency (2.5- fold with 75 μM Rib, and 2.8-fold with 100 μM Rib), relative to the population passaged in the absence of Rib (p<0.05 and p<0.005, respectively; χ^2^ test). A significant increase of nucleotide diversity was observed in the NS5A-coding region for populations treated with increasing concentrations of Rib (passage 4 populations, p<0.0017; Permutation test). Concerning the distribution of mutation types, again while the percentage of transitions relative to the total number of mutations did not vary as a result of Rib treatment (82.1%, 76.6% and 88.0% in the absence and presence of 75 μM and 100 μM Rib, respectively), the corresponding percentage of [(G→A)+(C→U)] transitions relative to the total number of mutations increased significantly [from 14.3% in the absence of Rib to 59.5% and 68.0% in the presence of 75 and 100 μM Rib, respectively (p<0.05 and p<0.01, respectively; χ^2^ test)] (Table 2 and Fig. 4b). The percentage of nonsynonymous mutations, the amino acid substitutions, and their probability of occurrence are given in Table S4. There is a very clear agreement between the mutant spectrum analyses of populations resulting from HCV infections in the presence of Rib at high and low MOI, indicating that Rib is mutagenic for HCVp0 replicating in hepatoma cells.

**Table 2:** Quasispecies analysis of NS5A genomic residues of HCVp0 populations passaged in the absence or presence of ribavirin^a^.

Ribavirin conc. (µM)^a^	Passage number	Number of nt analyzed (clones/haplotypes)^b^	Mutation frequency^c^	Nucleotide diversity^c^ π. 10^3^ (95% CI)
**0**	**4**	23,766 (17/13)	6.7×10^−4^	0.89 (0.72–1.19)
**0**	**5**	19,572 (14/8)	6.1×10^−4^	0.69 (0.42–1.12)
**75**	**4**	16,766 (12/12)	1.4×10^−3^	1.37 (0.96–2.05)
**75**	**5**	11,899 (9/8)	1.9×10^−3^	2.34 (1.76–3.06)
**100**	**4**	13,980 (10/10)	1.8×10^−3^	2.07 (1.75–2.44)

a The populations analyzed correspond to the infections at an initial MOI of 1 to 2 TCID_50_/cell described in Fig. 3a. Passage 5 in the infections with 100 μM Rib did not yield sufficient HCV RNA for analysis.The genomic residues analyzed are 6269 to 7666 of the NS5A-coding region. The HCV genome residue numbering corresponds to the JFH-1 genome (accession number #AB047639).

b The parenthesis indicates the number of clones analyzed, followed by the number of haplotypes (number of different RNA sequences); some clones did not contain the full length sequence; when the alignment of the sequenced region was correct such clones were entered in the calculation.

c Mutation frequency and nucleotide diversity are defined in Table 1 legend and Materials and Methods. Mutation types are summarized in Fig. 4b and their position in the HCV genome and deduced amino acid substitutions are given in Table S4.

An alternative sampling method of viral mutant spectra is provided by ultra-deep pyrosequencing (UDPS). We chose to determine the number of haplotypes and frequency of different transitions in six amplicons that spanned the entire NS5A-coding region of the HCVcc populations passaged 4 times in Huh-7.5 cells either in the absence or presence of 75 μM Rib. The results (Table 3, Fig. 4c and Table S5) indicate an increase in the number of different mutations, polymorphic sites, haplotypes and proportion of [(G→A)+(C→U)] in the populations passaged in the presence of Rib. Thus, UDPS also indicates a mutagenic activity of Rib on HCV.

**Table 3:** Ultra-deep pyrosequencing analysis of hepatitis C virus populations passaged in the absence or presence of ribavirin^a^.

		NS5A amplicon^b^					
Parameter	Ribavirin conc. (µM)^a^	A1	A2	A3	A4	A5	A6
		(6152–6454)	(6446–6767)	(6737–6954)	(6910–7252)	(7224–7550)	(7432–7725)
**Number of**	**0**	3	7	5	15	4	7
**different mutations**	**75**	18	25	15	35	22	24
**Number of**	**0**	3	7	5	14	4	6
**polymorfic sites**	**75**	18	24	15	34	21	24
**Number of**	**0**	4 (3/0)	8 (7/0)	6 (5/0)	16 (12/3)	4 (2/1)	8 (6/1)
**haplotypes** c	**75**	18 (15/2)	26 (24/1)	16 (14/1)	35 (31/3)	22 (19/2)	25 (21/3)
**[(G→A)+(C→U)]** d	**0**	2 (2/1)	0 (0/6)	0.25 (1/4)	0.50 (4/8)	1 (1/1)	0.33 (1/3)
**[(A→G)+(U→C)]**	**75**	3.5 (28/8)	2.83 (17/6)	6.5 (13/2)	1.45 (16/11)	13 (13/1)	3.75 (15/4)

a The populations analyzed correspond to the infections at an initial MOI of 1 to 2 TCID_50_/cell described in Fig. 3a.

b The HCV genome residue numbering corresponds to the JFH-1 genome (accession number #AB047639). Amplicon (A) length ranged between 216 and 339 nucleotides. The number of nucleotides sequenced was 2.1×10^6^ to 3.3×10^6^, and the number of reads on which the parameters were calculated was 10,000 for each amplicon. Procedures are described in Materials and Methods.

c In parenthesis the number of haplotypes with one and two mutations is given.

d In parenthesis the two terms of the ratio are given. Mutation types are summarized in Fig. 4c and their position in the HCV genome and deduced amino acid substitutions are given in Table S5.

### The effect of guanosine on the inhibitory and mutagenic activity of ribavirin. Alteration of nucleotide pools

**Figure 5:**
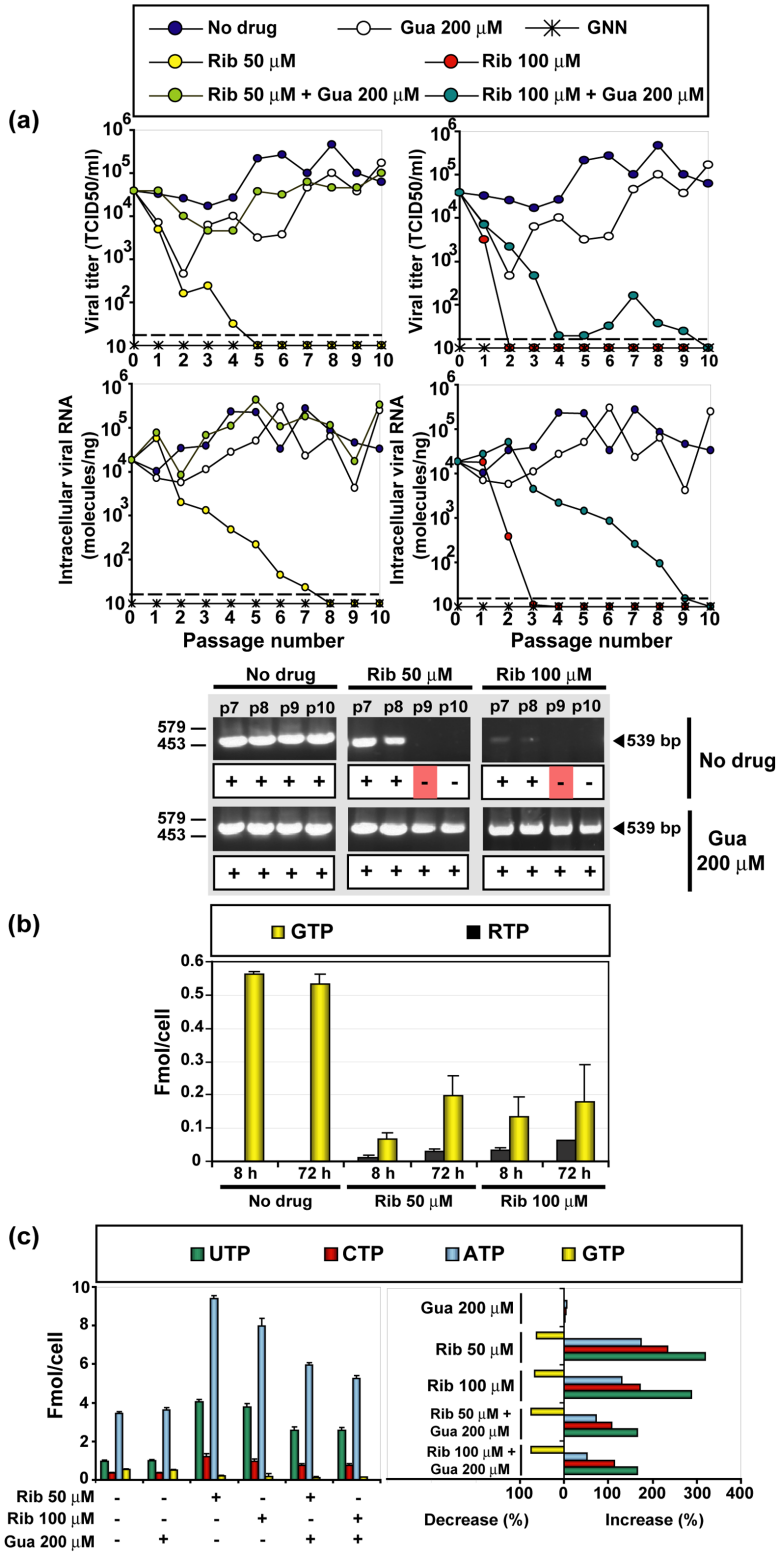
The effect of ribavirin (Rib) and guanosine (Gua) on HCV infectious progeny production and intracellular viral RNA and nucleotide pools. (a) Huh-7.5 reporter cells were infected with HCVp0 at a initial MOI of 0.1–0.2 TCID_50_/cell, in the absence or presence of the Rib and Gua concentrations indicated in the upper box. Infections with HCV GNN were carried out in parallel (negative control). The progeny form each infection was used to infect fresh cells, as described in Materials and Methods. Viral infectivity was determined in the cell culture supernatant (upper panels), and viral RNA quantitative RT-PCR in an extract of infected cells (lower panels). The discontinuous lines parallel to the abscissa indicates the limits of detection of infectivity and viral RNA. Below, RT-PCR amplification bands using a highly sensitive HCV-specific amplification protocol that yields a 539 bp fragment, using as template total intracellular RNA from the infection series and passage numbers indicated at the top of the corresponding lanes; +, −: presence or absence of amplification band. (b) Intracellular amount of GTP and RTP in Huh-7.5 reporter cells following 8 h or 72 h of exposure to the indicated amounts of Rib. (c) Left: intracellular amount of the four nucleoside-triphosphates, following 72 h of exposure to either Rib or Gua, as indicated at the bottom. Right: decrease or increase of nucleotide concentration as a result of exposure to Gua or Rib; note that the maximum decrease possible is 100%. Procedures are described in Materials and Methods.

Ribavirin is metabolized by cellular enzymes to produce the mono- di- and tri-phosphate nucleotide derivatives (RMP, RDP, RTP, respectively) [88], [104]. RMP is a competitive inhibitor of inosine monophosphate dehydrogenase (IMPDH), resulting in reduced intracellular GTP levels [88], [105], [106]. Guanosine can reverse the inhibitory effect of RMP on IMPDH [35], [60], [104], [107]. Therefore, we examined the effect of guanosine alone and guanosine plus Rib on HCV multiplication. Replication of HCVp0 in the presence of 200 μM guanosine resulted in a 10- to 100-fold decrease in HCV infectious progeny production and intracellular viral RNA, and the decrease diminished with passage number (Fig. 5a). Guanosine partially compensated the inhibition exerted by 50 μM Rib, but only minimally the inhibition by 100 μM Rib. In all cases the presence of guanosine delayed the Rib-mediated extinction of HCV. Only a minor reduction of mutant spectrum complexity (that did not reach statistical significance) was observed in the presence of guanosine at the two Rib concentrations tested (Table 4). Likewise, the Rib-mediated increase of nucleotide diversity and [(G→A)+(C→U)]/[(A→G)+(U→C)] ratio diminished only modestly in the presence of guanosine (Table 4 and Fig. 4d; the amino acid substitutions are listed in Table S6). To determine the effect of Rib treatment on the GTP level, the intracellular amount of GTP and RTP was measured in Huh-7.5 reporter cells at 8 h and 72 h after treatment with 50 μM and 100 μM Rib. The results (Fig. 5b) show a 3.0- to 8.5-fold reduction in the intracellular concentration of GTP. RTP reached maximum levels of 0.07 fmol/cell at 72 h of treatment with 100 μM Rib. In contrast to GTP, all other nucleotides increased their concentration as a result of Rib treatment, with maximum increases for the pyrimidine nucleotides (4.2-fold for UTP and 3.4-fold for CTP); the increase was partially reversed by the presence of guanosine (Fig. 5c). Thus, addition of guanosine to Huh-7.5 reporter cells did not restore the GTP depletion resulting from Rib addition. In view of these results, it was interesting to examine the effect of mycophenolic acid on HCV progeny production and mutagenesis.

**Table 4:** Quasispecies analysis of HCVp0 populations passaged in the absence or presence of ribavirin and guanosine^a^.

Ribavirin conc. (µM)^a^	Guanosine conc. (µM)^a^	Number of nt analyzed (clones/haplotypes)^b^	Mutation frequency^c^	Nucleotide diversity^c^ π. 10^3^ (95% CI)
**0**	**0**	29,704 (22/18)	7.7×10^−4^	1.16 (0.93–1.51)
**0**	**200**	30,493 (24/17)	8.5×10^−4^	1.36 (1.09–1.68)
**50**	**0**	23,766 (17/17)	1.4×10^−3^	1.54 (1.16–2.11)
**50**	**200**	35,979 (27/17)	9.2×10^−4^	1.39 (1.17–1.72)
**100**	**0**	21,396 (20/20)	2.0×10^−3^	2.43 (1.99–3.44)
**100**	**200**	38,125 (28/26)	1.7×10^−3^	2.36 (2.04–2.79)

a The populations analyzed correspond to the infections at an initial MOI of 0.1 to 0.2 TCID_50_/cell described in Fig. 5a. About 25% to 50% of clones analyzed did not contain the full length sequence expected from the primers used; when the alignment of the sequenced region was correct such clones were entered in the calculation. The HCV genome residue numbering corresponds to the JFH-1 genome (accession number #AB047639). The NS5A-coding region (nucleotides 6269 to 7666) was analyzed.

b The parenthesis indicates the number of clones analyzed, followed by the number of haplotypes (number of different RNA sequences); some clones did not contain the full length sequence; when the alignment of the sequenced region was correct such clones were entered in the calculation.

c Mutation frequency and nucleotide diversity are defined in Table 1 legend and Materials and Methods. Mutation types are summarized in Fig. 4d and their position in the HCV genome and deduced amino acid substitutions are given in Table S6.

### The effect of mycophenolic acid on hepatitis C virus progeny production and nucleotide pools

**Figure 6:**
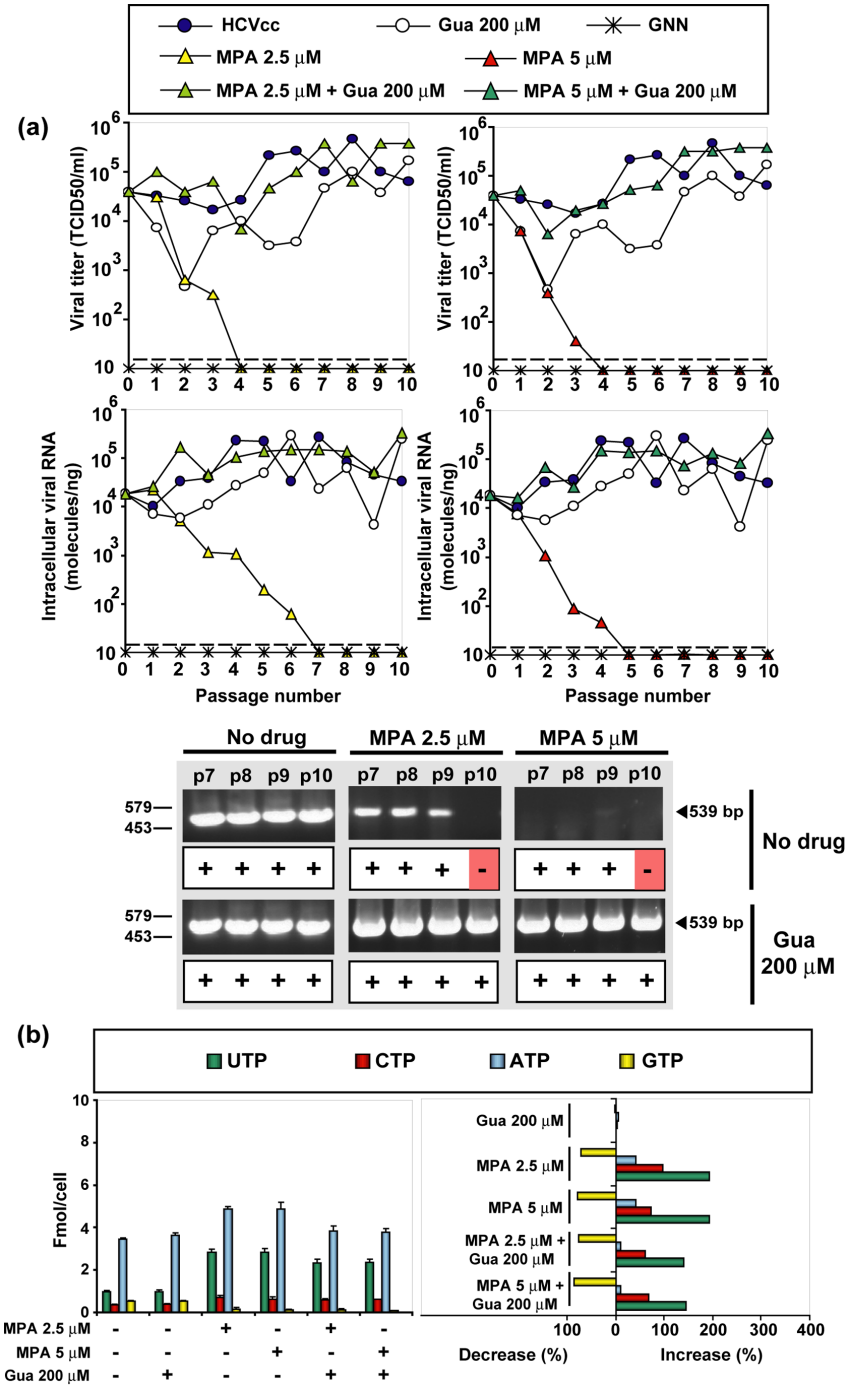
The effect of mycophenolic acid (MPA) and guanosine (Gua) on HCV infectious progeny production and intracellular nucleotide pools. (a) Huh-7.5 reporter cells were infected with HCVp0 at an initial MOI of 0.1–0.2 TCID_50_/cell, in the absence or presence of the MPA and Gua concentrations indicated in the upper box. Infections with HCV GNN were carried out in parallel (negative control). The progeny form each infection was used to infect fresh cells, as described in Materials and Methods. Viral infectivity was determined in the cell culture supernatant (upper panels), and viral RNA quantitative RT-PCR in an extract of infected cells (lower panels). The discontinuous lines parallel to the abscissa indicates the limits of detection of infectivity and viral RNA. Below, RT-PCR amplification bands using a highly sensitive HCV-specific amplification protocol that yields a 539 bp fragment, using as template total intracellular RNA from the infection series and passage numbers indicated at the top of the corresponding lanes; +, −: presence or absence of amplification band. (b) Left: Intracellular amount of the four nucleoside-triphosphates, following 72 h of exposure to either MPA or Gua, as indicated at the bottom. Right: decrease or increase of nucleotide concentration as a result of exposure to Gua or MPA; note that the maximum decrease possible is 100%. Procedures are described in Materials and Methods.

Mycophenolic acid is an inhibitor of IMPDH, which contrary to RTP or GTP cannot be incorporated into RNA [105], [108]–[110]. To explore whether depletion of GTP by itself (in the absence of an added mutagenic agent) could jeopardize infectious HCV progeny production, serial passages were carried out in the presence of mycophenolic acid in the absence or presence of guanosine. Mycophenolic acid produced a dramatic inhibition of HCV production that resulted in HCV extinction (Fig. 6a). Significantly, in the transition towards extinction, loss of infectivity preceded loss of intracellular viral RNA. The inhibitory effects of mycophenolic acid was almost fully reversed by guanosine, a reversal that was not observed previously with other hepatoma cells or HCV replicons [93], [111]. Depletion of GTP by mycophenolic acid was comparable to that produced by Rib, and also not reversed by guanosine addition, whereas the increase in the concentration of the other standard nucleotides was 1.5- to 4-fold lower (compare Figs. 5c and 6b). Passages in the presence of mycophenolic acid did not result in any significant increase of mutant spectrum complexity or mutational bias (Fig. 4d and Tables 5 and S7). Mycophenolic acid in the 2.5–5 μM range drove viral infectivity and RNA below the limit of detection. Furthermore, no HCV RNA could be amplified from the cell culture supernatant, using the sensitive RT-PCR protocol to diagnose extinction (Fig. 6a). The results suggest that depletion of GTP contributes to inhibition of HCV progeny production, without involving a mutagenic activity (see Discussion). The inhibitory effect of mycophenolic acid can drive HCV to extinction.

**Table 5:** Quasispecies analysis of HCVp0 populations passaged in the absence or presence of mycophenolic acid and guanosine^a^.

Mycophenolic acid conc. (µM)^a^	Guanosine conc. (µM)^a^	Number of nt analyzed (clones/haplotypes)^b^	Mutation frequency^c^	Nucleotide diversity^c^ π. 10^3^ (95% CI)
**0**	**0**	29,704 (22/18)	7.7×10^−4^	1.16 (0.93–1.51)
**0**	**200**	30,493 (24/17)	8.5×10^−4^	1.36 (1.09–1.68)
**5**	**0**	27,214 (23/15)	9.6×10^−4^	1.11 (0.81–1.67)
**5**	**200**	23,766 (17/12)	1.1×10^−3^	1.57 (0.90–2.45)

a The populations analyzed correspond to passage 3 (except MPA for 5 μM which is passage 2 because at passage 3 the amount of HCV RNA template was insufficient for the analysis) of infections at an initial MOI of 0.1–0.2 TCID_50_/cell described in Fig. 6a. The analyses for the control passages in the absence of mycophenolic acid are the same reported in Table 4. They are included here to facilitate comparisons. The HCV genome residue numbering corresponds to the JFH-1 genome (accession number #AB047639). The NS5A-coding region (nucleotides 6269 to 7666) was analyzed.

b The parenthesis indicates the number of clones analyzed, followed by the number of haplotypes (number of different RNA sequences); some clones did not contain the full length sequence; when the alignment of the sequenced region was correct such clones were entered in the calculation.

c Mutation frequency and nucleotide diversity are defined in Table 1 legend and Materials and Methods. Mutation types are summarized in Fig. 4d and their position in the HCV genome and deduced amino acid substitutions are given in Table S7.

### Probing features of lethal mutagenesis in the extinction of hepatitis C virus by ribavirin and mycophenolic acid

**Figure 7:**
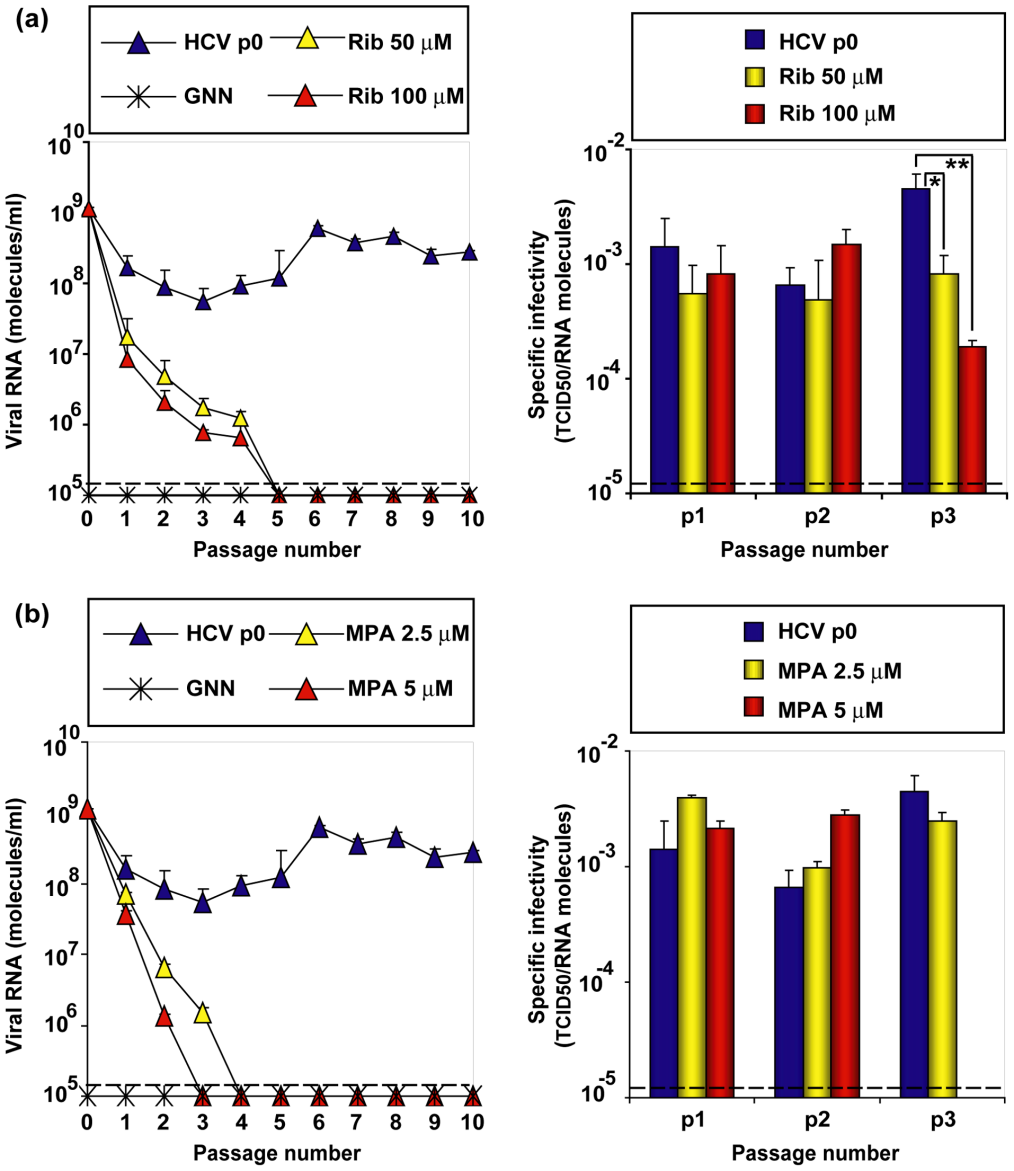
Effect of ribavirin (Rib) and mycophenolic acid (MPA) on the specific infectivity of HCV. (a) Huh-7.5 reporter cells were infected with HCVp0 at an initial MOI of 0.1–0.2 TCID50/cell, in the absence or presence of the Rib concentrations indicated in the upper box; infection with GNN was used as negative control. The experiment is the one shown in Fig. 3(b). At different passages, extracellular viral RNA was measured by quantitative RT-PCR (left panel). Specific infectivities (right panel) were calculated with the infectivities given in Fig. 3b and the RNA concentrations indicated in the left panel. The discontinuous horizontal line indicates the limit of detection. Statistically significant differences are indicated by one (p<0.001) or two (p<0.0001) asterisks (one way analysis of variance as detailed in Materials Methods). (b) Infections as in (a), in the absence or presence of MPA concentrations indicated in the boxes above the panels. The experiment is the one shown in Fig. 5(a). At different passages, extracellular viral RNA was measured by quantitative RT-PCR (left panel). Specific infectivities (right panel) were calculated with the infectivities given in Fig. 5(a) and the RNA concentrations indicated in the left panel. The discontinuous horizontal line indicates the limit of detection. Differences were not statistically significant. Procedures are described in Materials and Methods.

Mutagenesis-driven extinction of FMDV and LCMV occurred with 10^2^-to 10^3^-fold decreases of specific infectivity (the ratio between viral infectivity and the amount of genomic viral RNA) and without any detectable variation in the consensus genomic nucleotide sequence [42], [112], [113]. These features are a hallmark of virus extinction by lethal mutagenesis [reviewed in [114], [115]]. Extinction by Rib occurred with a 5-fold to 23-fold decrease of specific infectivity, as quantified by infectivity and viral RNA in samples of the cell culture supernatants (Fig. 7a). In contrast, no variation in specific infectivity was observed during mycophenolic acid-mediated extinction (Fig. 7b). In both cases the consensus sequence remained invariant. Thus, Rib-mediated, but not mycophenolic acid-mediated, HCV extinction displayed the hallmarks of lethal mutagenesis. In conclusion, we have provided evidence that Rib alters the mutant spectrum composition of HCV quasispecies replicating in hepatoma cells in a manner compatible with a mutagenic activity of Rib.

## Discussion

Lethal mutagenesis is gradually finding its way as a potential antiviral therapy with reduced probability of selection of escape mutants [54], [116], [117], and with prospects of applying the same principles to anti-cancer therapy [118]–[120]. The mechanism by which Rib exerts its antiviral activity is still a debated issue since multiple, unrelated modes of action have been observed with different viruses [59], [106], [121], and more than one mechanism may be operating on a given virus. Recent studies of antiviral designs based on lethal mutagenesis render important a clarification of whether mutagenesis of the viral genome occurs in the course of an antiviral treatment. Indeed, a potential advantage of a sequential inhibitor-mutagen administration over the corresponding combination, necessitates that a mutagenic agent be part of the therapy [95]–[97]. We are currently extending to HCV our previous approach of testing the efficacy of alternative administration protocols [reviewed in [114]]. Rib is ideally suited for the investigations on lethal mutagenesis of HCV because it is a component of SOC treatment. In the present investigation, the mutagenic activity of Rib on HCV replicating in Huh-7.5 reporter cells has been documented by statistically significant increases of mutant spectrum complexity and an increase of G→A and C→U transitions. In the NS5A-coding region, UDPS identified a total of 38 and 127 different mutations in the populations passaged in the absence and presence of Rib, respectively. The corresponding numbers identified by molecular cloning and Sanger sequencing were 28 and 46, respectively. Of the total number of different mutations scored, only 5 and 12, in the absence and presence of Rib, respectively, were common to Sanger sequencing and UDPS (Tables S4 and S5). Thus, despite sampling different sequences, both procedures provide evidence of a mutagenic activity of Rib on HCV. Other activities of Rib in Huh-7.5 cells may contribute to the anti-HCV action of Rib. It must be indicated, however, that solely a change in mutant spectrum composition does not provide evidence of a mutagenic activity, since a drug may alter the relative fitness of viral subpopulations. This might cause a variation in the broadness of the mutant spectrum. In our case, however, it seems unlikely that such mutant spectrum reorganization would lead to a mutational bias in favor of G→A and C→U transitions in the absence of a mutagenic activity associated with Rib treatment.

Depletion of intracellular GTP levels does not account for the majority of Rib-mediated mutagenesis, since no mutagenesis was observed under the same depletion as a result of treatment with mycophenolic acid. This immunosuppressant and some of its prodrugs have proven effective as inhibitors of HCV multiplication in cell culture and *in vivo* [93], [111], [122], [123]. Significantly, extinction by the non-mutagenic mycophenolic acid did not alter the specific infectivity of HCV and, with our quantification procedures infectivity was still detectable when extracellular HCV RNA was not. This is in contrast to the decrease in specific infectivity associated with Rib-mediated extinction, a hallmark of lethal mutagenesis [35], [42], [57], [61], [62], [97], [113] (Fig. 7).

It is not clear whether the inhibition of HCV progeny production by Rib is directly linked to its mutagenic activity or to some other effect on the virus or the host cell, independent of its mutagenic activity. The difference in compensatory activity of guanosine on the inhibition produced by 50 μM and 100 μM Rib does not appear to be related to variations in intracellular GTP. Rather, Rib produced a considerable increase in intracellular ATP, CTP and UTP that was only partially compensated by guanosine addition. Concentrations of mycophenolic acid that depleted GTP to a similar extent than Rib led to a less accentuated increase of ATP, CTP and UTP. Thus, imbalances in the levels of the standard nucleotides might contribute to the inhibition produced by Rib. Modifications of hepatocyte gene transcription may affect completion of the virus life cycle [124]. ATP – which underwent a 2-fold higher increase upon treatment with Rib than with mycophenolic acid (Figs. 5 and 6) – is recruited in HCV-infected hepatocytes where it co-localizes with non-structural HCV proteins in cytoplasmic structures; the ATP concentration is about 5-fold higher at these sites than elsewhere in the cell [125]. ATP levels may modify the activity of the ATP-dependent molecular chaperon HSP90, which plays a critical role in HCV NS5B phosphorylation [126]. Thus, formation and activity of protein complexes involved in HCV replication may be more sensitive than mutagenesis to nucleotide pool imbalances. It cannot be excluded, however, that mycophenolic acid displays some anti-HCV activity other than the one derived from inhibition of IMPDH.

The addition of guanosine does not have the same effect on different viruses replicating in the presence of Rib. In a persistent infection of FMDV in BHK-21 cells, guanosine partially reversed the antiviral but not the mutagenic activity of Rib, but it compensated a modest mutagenic activity induced by mycophenolic acid [35]. Guanosine greatly abolished the inhibition of LCMV progeny production evoked by ribavirin or mycophenolic acid [61], and it reversed the non-mutagenic inhibitory effect of Rib exerted on porcine nidoviruses [63]. These differences may lie in the viral polymerases (or their replication complexes) which might vary in affinity for RTP and in misincorporations under biased nucleotide concentrations, as well as in metabolic cell alterations associated with the infection. The types of mutations evoked by Rib, as well as the proportion of the nucleotides found at the 5′ side (22% A, 26% G, 8% U, and 44% C) and 3′side (18% A, 31% G, 12% U, 39% C) of the mutation site in the NS5A region – values that mirror approximately the base composition of this genomic region – render unlikely the participation of the known ADAR activities on Rib-induced mutagenesis [127], [128].

The documented mutagenic activity of Rib on HCV opens the way to pursuing research on alternative anti-HCV protocols [49], [95]–[97], and such experiments are now in progress. A related issue is to what extent Rib mutagenesis participates in the benefits of SOC. One argument against a mutagenic activity of Rib during therapy is that the intracellular hepatic levels of Rib are unlikely to reach those used in cell culture (50 μM to 100 μM, which are 3- to 100-fold higher than those considered achievable during therapy *in vivo* [129]). It must be considered, however, that a broad range of Rib concentrations in human serum and in organs targeted by Rib has been reported [130]–[137]. Moreover, neither the effective RTP concentration in liver cells and at the membrane-bound HCV replication complexes, nor RMP and RDP levels – which would be informative of the potential activities of Rib metabolites during SOC – have been measured. Therefore, it is not possible to relate results in hepatoma cells in culture with those *in vivo* either to support or to dismiss a mutagenic activity of Rib on HCV *in vivo*.

Several possibilities can account for conflicting results regarding the mutagenic character of Rib for HCV: (i) A limited number of HCV RNA replication rounds in the presence of Rib may be insufficient to detect an increase in mutant spectrum complexity. We have tested this possibility by carrying out a mutant spectrum analysis of HCV after a single infection of 4×10^6^ Huh-7.5 reporter cells with HCVp0 at a MOI of 0.2 TCID_50_/cell and 50 μM Rib and 100 μM Rib. Neither the increase in mutation frequency nor that of G→A and C→U transitions reached statistical significance (A.M. Ortega-Prieto et al., unpublished results). (ii) Rib can produce transient expansions of mutant spectrum complexity [102], [138], so that a mutational activity might be missed depending on the time of sampling of the mutant spectrum. (iii) If quantifications of template HCV RNA are not performed prior to RT-PCR amplifications intended for molecular cloning and Sanger sequencing or for UDPS, the repertoire of genomes screened might not reflect the repertoire present in the natural sample. Both, molecular cloning and sequencing and deep-sequencing methods are only sampling methods to approach the complex reality of a viral mutant spectrum. Low viral loads such as those produced by Rib will tend to underestimate the mutant spectrum complexity unless the amounts of template are normalized prior to RT-PCR amplification. (iv) In combination therapies, the interplay between an inhibitor and a mutagenic agent may affect the timing in which an increase in mutant spectrum complexity can be observed [95], [96]. It may be significant that evidence of a mutagenic activity *in vivo* has been obtained during Rib monotherapy [77]. These possible sources of bias apply to determinations of mutant spectrum complexity both in cell culture and *in vivo*, and should be evaluated in comparing results from different studies.

An analysis of the molecular events that underlie the transition towards HCV extinction has indicated an apparently stochastic persistence of HCV RNA despite loss of HCV infectivity. There have been clinical descriptions of presence of HCV RNA in patients displaying a sustained response to treatment [139]–[141]. The nature of this persistent HCV RNA in hepatocytes has not been studied, but its presence cannot be dismissed because it might correspond to defective HCV RNAs. A class of defective viral genomes termed defectors participate in lethal mutagenesis and result in loss of infectivity preceding loss of viral RNA [42], [114]. The possible involvement of lethal defectors in HCV extinction and the biological significance of HCV RNA remnants following an effective treatment deserve further investigation.

## Materials and Methods

### Cells and viruses

The origin of Huh 7.5, Huh 7-Lunet, Huh-7.5 reporter cell lines and procedures for cell growth in Dulbecco's modification of Eagle's medium (DMEM), have been previously described [142], [143]. Infected and uninfected cells were cultured at 37°C and 5% CO_2_. The viruses used in the experiments are HCVcc [Jc1FLAG2(p7-nsGluc2A)] (a chimera of J6 and JFH-1 from genotype 2a) and GNN [GNNFLAG2(p7-nsGluc2A)] (carrying a mutation in the NS5B RNA-dependent RNA polymerase rendering it replication-defective) [144]. To control for the absence of contamination the supernatants of mock-infected cells maintained in parallel with the infected cultures were titrated; no infectivity in the mock-infected cultures was detected in any of the experiments.

### Production of viral progeny and titration of infectivity

The procedures used to prepare the initial virus stock HCVp0 and for serial infections of the hepatoma Huh-7.5 cells have been previously described [12]. Briefly, Huh-7-Lunet cells were electroporated with 10 μg of the infectious transcript of HCVcc (Jc1 or the negative control GNN) (260 volts, 950 μF). Electroporated cells were then passaged every 3–4 days without allowing the cells to become confluent, passages were continued until 30 days post-electroporation, and the cell culture supernatants were pooled. The virus was then concentrated 20 times using 10,000 MWCO spin columns (Millipore) as instructed by the manufacturer, and stored in aliquots (at −70°C). To increase virus infectivity, Huh-7.5 reporter cells were infected with concentrated virus stocks at a MOI of 0.5 TCID_50_/cell, and the cells were passaged to obtain the working viral stock HCVp0 [12]. For titration of HCV infectivity, serially diluted cell culture supernatants were applied to Huh-7.5 cells and 3 days post-infection the cells were washed with PBS, fixed with ice-cold methanol, and stained using anti-NS5A monoclonal antibody 9E10, as previously described [12], [145].

### Treatment with ribavirin, guanosine and mycophenolic acid

Solutions of Rib (Sigma), guanosine (Sigma) and mycophenolic acid (Sigma) were prepared at concentrations of 100 mM, 200 μM and 50 mg/ml in PBS, DMEM and methanol, respectively. They were sterilized by filtration, and stored at –70°C. Prior to use, the stock solutions were diluted in DMEM to reach the desired concentration. Huh-7.5 reporter cells were pretreated with the appropriate drug concentrations during 16 h prior to infection. Then, 4×10^6^ cells were infected with 8×10^5^ TCID_50_ of HCVp0; the adsorption time was 5 h, and the infection continued for 72 to 96 h in the presence of Rib. For successive viral passages, 4×10^6^ Huh-7.5 reporter cells were infected with 2.5 ml of the supernatant from the previous infection; the MOI ranged between 0.1 and 10^−4^ TCID_50_/cell; each MOI can be calculated from the infectivity values given for each experiment. In some cases the initial MOI was 1 to 2 TCID_50_/cell, as detailed in the corresponding experiments.

### Toxicity test

The CC_50_ of Rib, and mycophenolic acid was measured by seeding 96-well plates with Huh-7.5 cells to 70% confluence and exposing the cells to up to 250 μM Rib and 100 μM mycophenolic acid for 72 hours. MTT [3-(4,5-dimethylthiazol-2-yl)-2,5-diphenyltetrazolium bromide] was added to each well at a final concentration of 500 μg/ml; 4 h later crystals were dissolved in 100 μl of DMSO and the O.D. measured at 550 nm; 50% cytotoxicity was calculated from four different determinations as previously described [144].

### Inhibitory concentration

The IC_50_ of Rib, and mycophenolic acid was calculated relative to the untreated controls (defined as 100% infectivity), as described previously [57]; determinations were carried out in triplicate.

### Nucleotide pool analysis

The procedure used has been previously described [146], [147]. Huh-7.5 cells (2×10^6^ cells) either untreated or treated with the drugs indicated for each experiment were washed with PBS and incubated on ice for 10 min with 900 μl of 0.6 M trichloroacetic acid. A precooled mixture of 720 µl of Uvasol® (1,1,2-trichlorotrifluoroethane, Sigma) and 180 µl of Tri-n-octylamine (Sigma) was added to 900 µl of the supernatant, vortexed for 10 s and centrifuged 30 s at 12,000 g at 4°C and stored at −80°C until further analysis. Nucleotides were separated in a Partisil 10 SAX analytical column (4.6 mm×250 mm) (Whatman) with a Partisil 10 SAX guard cartridge column (4.6×30 mm) (Capital HPLC). Each sample (100 µl) was injected into an Alliance 2695 HPLC system connected to a 2996 photodiode array detector (Waters); the eluent flow rate was 0.8 ml/min, and the nucleotides were detected at a wavelength of 254 nm, except Rib that was detected at 220 nm. Prior to injections, the column was equilibrated with 60 ml of 7 mM NH_4_H_2_PO_4_, pH 3.8 (buffer A). The separation program started with 22.5 min of an isocratic period with buffer A followed by a linear gradient of 112.5 min to the high concentration buffer 250 mM NH_4_H_2_PO_4_, 500 mM KCl, pH 4.5 (buffer B) and a final isocratic period of 37.5 min with buffer B. Prior to sample analysis, 50 µl of 20 pmol/µl UTP, CTP, ATP and GTP, and 80 pmol/µl Ribavirin (Jena Bioscience), were separated to create a processing method using the Waters Empower™ Chromatography Data Software. Determinations were carried out with two independent biological samples, each one in triplicate. The amount of each nucleotide in cell extracts was normalised relative to the number of cells.

### RNA extraction, cDNA synthesis, PCR amplification and nucleotide sequencing

Intracellular viral RNA was extracted from infected cells using the Qiagen RNeasy kit according to the manufacturer's instructions (Qiagen, Valencia, CA, USA). RT-PCR amplification was carried out using AccuScript (Agilent), as specified by the manufacturers. Several genomic regions were amplified using specific oligonucleotides as primers (Table S8). Nucleotide sequences of genomic HCV RNA were determined using the 23 ABI 3730XLS sequencer. To evaluate the complexity of mutant spectra, HCV RNA was extracted as described above and subjected to RT-PCR to amplify the E2-, NS5A- and NS5B-coding regions as previously described [12]. Amplification products were analyzed by agarose gel electrophoresis using HindIII-digested Ф-29 DNA as molar mass standards. Negative controls (amplifications in the absence of RNA) were included in parallel to ascertain absence of contamination by template nucleic acids. To ensure an excess of template in the RT-PCR amplifications for quasispecies analysis, and to avoid complexity biases due to redundant amplifications of the same initial RNA templates, amplifications were carried out with template preparations diluted 1∶10, 1∶100 and 1∶1000; only when the 1∶100 diluted template produced a visible DNA band was molecular cloning pursued using the DNA amplified from undiluted template [35]. Controls to ascertain that mutation frequencies were not affected by the basal error rate during amplification have been previously described [148].

For the UDPS analysis (GS-FLX platform, 454 Life Sciences-Roche), RT-PCR was performed using Accuscript (Agilent). To cover the complete NS5A region, and considering that the GS-FLX Titanium chemistry allows sequencing fragments of 400–500 nucleotides, this genomic region was divided into six overlapping amplicons, and amplification products obtained using specific primers (Table S8). To minimize the errors due to PCR reactions, RT-PCR amplifications were performed in triplicate and mixed equimolarly prior to the analysis. Then, PCR products were purified (Ampure Beads), quantified (Pico Green Assay), and analyzed for quality (Bioanalyzer) prior to the UDPS procedure. Negative controls (without template RNA) were run in parallel to ascertain absence of contamination with undesired templates. Data analysis was based on the fasta files obtained from the 454 GS system software which includes quality controls to guarantee the integrity of the amplicons. Data processing was done on the open source R environment [149], using Bioconductor [150] and the Biostrings library [151] for pattern matching, sequence alignment and functions developed for this purpose. Two levels of errors were considered: a background level of noise that affected both strands, and at a higher level which was neighborhood and structure-dependent, and therefore strand-specific. For reads data processing a demultiplexing step where the reads were assigned to samples and amplicons was followed by a quality filter and repair step, and by the intersection of haplotypes in the forward and reverse strands, and an abundance filter for these consensus haplotypes above 0.25% [152]. Accuracy validations were performed using published procedures [150], [153]–[155]; the same conclusions on Rib mutagenesis were obtained with a cut-off value of 1.0%.

### Population complexity

Population complexity was measured by determining mutation frequency, number of polymorphic sites and haplotypes as described in the corresponding tables. Nucleotide diversity (π) (average number of nucleotide differences per site between any two RNA sequences of the same mutant spectrum and genomic region) was calculated according to the formula π = n (n−1)/2 Σ_ij_ π_ij_ (i<j) where n is the number of clones, and π_ij_ is the number of different nucleotides between the pair of sequences ij divided by the sequence length in nucleotides [156]. Confidence intervals for nucleotide diversities were calculated using bias-corrected bootstrap resampling method.

### Statistical analyses

The statistical significance of differences between mutation frequencies or repertoires of mutation types was evaluated by the chi-square test (χ^2^ test). To determine the significance of increases of nucleotide diversity associated with drug treatments, permutation tests were carried out on the regression slope of nucleotide diversity as a function of drug concentration, with 10,000 permutations per test. Briefly, sequences were randomly assigned to one of the possible groups, keeping the group size as in the sample under analysis. For each permutation, a linear regression of the nucleotide diversity versus drug concentration was obtained, and the value of the slope was stored. In this manner, the distribution of the regression slope was obtained for the case in which differences in nucleotide diversity were due solely to random sampling. Then, a p-value for the observed slope was calculated as the number of times that an equal or higher slope than the observed was found in the null distribution divided by the number of permutations. Both, p-values from permutations tests and bias-corrected bootstrap confidence intervals were obtained using ad hoc scripts developed for MATLAB® (code available upon request).

To probe statistical significance of the differences in specific infectivity, one-way analysis of the variance was performed with statistical package SPSS 13.0 (SPSS, Inc.). For multiple comparisons, Bonferroni's correction was applied. The data are presented as mean values and standard deviations.

### Quantification of HCV RNA

Real time quantitative RT-PCR was carried out using the Light Cycler RNA Master SYBR Green I kit (Roche), according to the manufacturer's instructions, as previously described [157]. The 5′-UTR non-coding region of the HCV genome was amplified using as primers oligonucleotide HCV-5UTR-F2 (5′- TGAGGAACTACTGTCTTCACGCAGAAAG; sense orientation; the 5′ nucleotide corresponds to genomic residue 47), and oligonucleotide HCV-5UTR-R2 (5′- TGCTCATGGTGCACGGTCTACGAG; antisense orientation; the 5′ nucleotide corresponds to genomic residue 347). Quantification was relative to a standard curve obtained with known amounts of HCV RNA, synthesized by *in vitro* transcription of plasmid GNN DNA. The specificity of the reaction was monitored by determining the denaturation curve of the amplified DNAs. Negative controls (without template RNA and RNA from mock-infected cells) were run in parallel with each amplification reaction, to ascertain absence of contamination with undesired templates.

### Assessment of HCV extinction

We have taken as criteria to consider HCV extinct those previously described for lethal mutagenesis of FMDV [48], [49]. HCV was considered extinct when no virus infectivity was detected and no viral RNA was amplified using a sensitive RT-PCR amplification protocol, either from the supernatant of the cell culture that contains the putatively extinguished virus, or following 3 blind passages of the cell culture supernatants using Huh-7.5 reporter cells in the absence of any drug. The highly sensitive RT-PCR consists in the amplification using the primers JC1-NS5A F1 and JC1-NS5A R1 (Table S8). It should be noted that infectivity below the level of detection did not necessarily imply extinction according to these criteria, and this is indicated in the corresponding results.

## Supporting Information

Table S1 **Mutations, corresponding amino acid and point accepted mutation (PAM) of the E2-coding region in the mutant spectra HCV p3 passaged in the absence or presence of ribavirin (Rib).**
(DOC)

Table S2 **Mutations, corresponding amino acid and point accepted mutation (PAM) of the NS5A-coding region in the mutant spectra HCV p3 passaged in in the absence or presence of ribavirin (Rib).**
(DOC)

Table S3 **Mutations, corresponding amino acid and point accepted mutation (PAM) of the NS5B-coding region in the mutant spectra HCV p3 passaged in the absence or presence of ribavirin (Rib).**
(DOC)

Table S4 **Mutations, corresponding amino acid and point accepted mutation (PAM) of the NS5A-coding region in the mutant spectra HCV p4 and p5 passaged in the absence or presence of ribavirin (Rib).**
(DOC)

Table S5 **Mutations, corresponding amino acid and point accepted mutation (PAM) of the NS5A-coding region in the mutant spectra HCV p4 passaged in the absence or presence of ribavirin (Rib) analyzed by ultra deep sequencing.**
(DOC)

Table S6 **Mutations, corresponding amino acid and point accepted mutation (PAM) of the NS5A-coding region in the mutant spectra HCV p3 passaged in the absence or presence of ribavirin (Rib) and guanosine (Gua).**
(DOC)

Table S7 **Mutations, corresponding amino acid and point accepted mutation (PAM) of the NS5A-coding region in the mutant spectra HCV p3 passaged in the absence or presence of guanosine (Gua) and/or mycophenolic acid (MPA).**
(DOC)

Table S8 **Oligonucleotides used to amplify and sequence the HCV genomes.**
(DOC)

## References

[1] Ploss A, Dubuisson J (2012) New advances in the molecular biology of hepatitis C virus infection: towards the identification of new treatment targets. Gut 61 Suppl 1i25–35. 22504917 10.1136/gutjnl-2012-302048

[2] Deuffic-Burban S, Poynard T, Sulkowski MS, Wong JB (2007) Estimating the future health burden of chronic hepatitis C and human immunodeficiency virus infections in the United States. J Viral Hepat 14: 107–115. 17244250 10.1111/j.1365-2893.2006.00785.x

[3] Quer J, Martell M, Rodriguez A, Bosch A, Jardi R, et al.. (2008) The impact of Rapid Evolution of Hepatitis Viruses, p. 303–350. In Origin and Evolution of Viruses. Domingo, E., Parrish, C. and Holland, J.J. (eds.). Elsevier, Oxford.

[4] Williams R (2006) Global challenges in liver disease. Hepatology 44: 521–526. 16941687 10.1002/hep.21347

[5] Farci P (2011) New insights into the HCV quasispecies and compartmentalization. Sem Liver Disease: 356–374. 10.1055/s-0031-1297925 22189976

[6] Martell M, Esteban JI, Quer J, Genesca J, Weiner A, et al (1992) Hepatitis C virus (HCV) circulates as a population of different but closely related genomes: quasispecies nature of HCV genome distribution. J Virol 66: 3225–3229. 1313927 10.1128/jvi.66.5.3225-3229.1992 PMC241092

[7] Pawlotsky JM (2006) Hepatitis C virus population dynamics during infection. Current Topics in Microbiol and Immunol 299: 261–284. 10.1007/3-540-26397-7_9 16568902

[8] Fried MW, Shiffman ML, Reddy KR, Smith C, Marinos G, et al (2002) Peginterferon alfa-2a plus ribavirin for chronic hepatitis C virus infection. N Engl J Med 347: 975–982. 12324553 10.1056/NEJMoa020047

[9] Ghany MG, Strader DB, Thomas DL, Seeff LB (2009) Diagnosis, management, and treatment of hepatitis C: an update. Hepatology 49: 1335–1374. 19330875 10.1002/hep.22759 PMC7477893

[10] Chevaliez S, Pawlotsky JM (2007) Interferon-based therapy of hepatitis C. Adv Drug Deliv Rev. 59: 1222–1241. 10.1016/j.addr.2007.07.002 17869375

[11] Hadziyannis SJ, Sette H Jr, Morgan TR, Balan V, Diago M, et al (2004) Peginterferon-alpha2a and ribavirin combination therapy in chronic hepatitis C: a randomized study of treatment duration and ribavirin dose. Ann Intern Med 140: 346–355. 14996676 10.7326/0003-4819-140-5-200403020-00010

[12] Perales C, Beach NM, Gallego I, Soria ME, Quer J, et al (2013) Response of hepatitis C virus to long-term passage in the presence of alpha interferon. Multiple mutations and a common phenotype. J Virol 87: 7593–7607. 23637397 10.1128/JVI.02824-12 PMC3700284

[13] Lange CM, Sarrazin C, Zeuzem S (2010) Review article: specifically targeted anti-viral therapy for hepatitis C – a new era in therapy. Aliment Pharmacol Ther 32: 14–28. 20374226 10.1111/j.1365-2036.2010.04317.x

[14] Kwong AD, Najera I, Bechtel J, Bowden S, Fitzgibbon J, et al.. (2011) Sequence and Phenotypic Analysis for Resistance Monitoring in Hepatitis C Virus Drug Development: Recommendations From the HCV DRAG. Gastroenterology. 10.1053/j.gastro.2011.01.029 21255574

[15] Feld JJ (2012) Is there a role for ribavirin in the era of hepatitis C virus direct-acting antivirals? Gastroenterology 142: 1356–1359. 22537443 10.1053/j.gastro.2011.12.064

[16] Thomas E, Ghany MG, Liang TJ (2012) The application and mechanism of action of ribavirin in therapy of hepatitis C. Antivir Chem Chemother. 23: 1–12. 10.3851/IMP2125 PMC6271563 22592135

[17] Gelman MA, Glenn JS (2011) Mixing the right hepatitis C inhibitor cocktail. Trends Mol Med 17: 34–46. 21106440 10.1016/j.molmed.2010.10.005 PMC3085044

[18] Cubero M, Esteban JI, Otero T, Sauleda S, Bes M, et al (2008) Naturally occurring NS3-protease-inhibitor resistant mutant A156T in the liver of an untreated chronic hepatitis C patient. Virology 370: 237–245. 18006035 10.1016/j.virol.2007.10.006

[19] Domingo E (2003) Quasispecies and the development of new antiviral strategies. Progress in Drug Res 60: 133–158. 10.1007/978-3-0348-8012-1_4 12790341

[20] Richman DD, editor (1996) Antiviral Drug Resistance. New York: John Wiley and Sons Inc.

[21] Mas A, Lopez-Galindez C, Cacho I, Gomez J, Martinez MA (2010) Unfinished stories on viral quasispecies and Darwinian views of evolution. J Mol Biol 397: 865–877. 20152841 10.1016/j.jmb.2010.02.005

[22] Domingo E, Biebricher C, Eigen M, Holland JJ (2001) Quasispecies and RNA Virus Evolution: Principles and Consequences. Austin: Landes Bioscience.

[23] Kuntzen T, Timm J, Berical A, Lennon N, Berlin AM, et al (2008) Naturally occurring dominant resistance mutations to hepatitis C virus protease and polymerase inhibitors in treatment-naive patients. Hepatology 48: 1769–1778. 19026009 10.1002/hep.22549 PMC2645896

[24] Sarrazin C, Zeuzem S (2010) Resistance to direct antiviral agents in patients with hepatitis C virus infection. Gastroenterology 138: 447–462. 20006612 10.1053/j.gastro.2009.11.055

[25] Margeridon-Thermet S, Shafer RW (2010) Comparison of the Mechanisms of Drug Resistance among HIV, Hepatitis B, and Hepatitis C. Viruses. 2: 2696–2739. 10.3390/v2122696 PMC3020796 21243082

[26] Eigen M (2002) Error catastrophe and antiviral strategy. Proc Natl Acad Sci USA 99: 13374–13376. 12370416 10.1073/pnas.212514799 PMC129678

[27] Schuster P, Stadler PF (2008) Early Replicons: Origin and Evolution. In: Domingo E, Parrish CR, Holland JJ, editors. Origin and Evolution of Viruses 2^nd^ edition. Oxford: Elsevier. 1–42.

[28] Bull JJ, Sannjuán Ra, Wilke CO (2008) Lethal mutagenesis. In: Origin and Evolution of Viruses: (Domingo, E, Parrish, C and Holland, J.J. eds.). Elsevier, 207–218.

[29] Ochoa G (2006) Error thresholds in genetic algorithms. Evol Comput 14: 157–182. 16831105 10.1162/evco.2006.14.2.157

[30] Alves D, Fontanari JF (1998) Error threshold in finite populations. Physical Review E 57: 7008–7013.

[31] Takeuchi N, Hogeweg P (2007) Error-threshold exists in fitness landscapes with lethal mutants. BMC Evol Biol 7: 15; author reply 15. 10.1186/1471-2148-7-15 PMC1805495 17286853

[32] Chen P, Shakhnovich EI (2009) Lethal mutagenesis in viruses and bacteria. Genetics 183: 639–650. 19620390 10.1534/genetics.109.106492 PMC2766323

[33] Schuster P (2011) Lethal mutagenesis, error thresholds, and the fight against viruses: Rigorous modeling is facilitated by a firm physical background. Complexity. 5–9.

[34] Wylie CS, Shakhnovich EI (2012) Mutation induced extinction in finite populations: lethal mutagenesis and lethal isolation. PLoS Comput Biol 8: e1002609. 22876168 10.1371/journal.pcbi.1002609 PMC3410861

[35] Airaksinen A, Pariente N, Menendez-Arias L, Domingo E (2003) Curing of foot-and-mouth disease virus from persistently infected cells by ribavirin involves enhanced mutagenesis. Virology 311: 339–349. 12842623 10.1016/s0042-6822(03)00144-2

[36] Anderson JP, Daifuku R, Loeb LA (2004) Viral error catastrophe by mutagenic nucleosides. Annu Rev Microbiol 58: 183–205. 15487935 10.1146/annurev.micro.58.030603.123649

[37] Crotty S, Cameron CE, Andino R (2001) RNA virus error catastrophe: direct molecular test by using ribavirin. Proc Natl Acad Sci USA 98: 6895–6900. 11371613 10.1073/pnas.111085598 PMC34449

[38] Day CW, Smee DF, Julander JG, Yamshchikov VF, Sidwell RW, et al (2005) Error-prone replication of West Nile virus caused by ribavirin. Antiviral Res 67: 38–45. 15919121 10.1016/j.antiviral.2005.04.002

[39] Domingo E, (ed) ( Virus entry into error catastrophe as a new antiviral strategy. Virus Res 107: 115–228.

[40] Graci JD, Cameron CE (2008) Therapeutically targeting RNA viruses via lethal mutagenesis. Future Virol 3: 553–566. 19727424 10.2217/17460794.3.6.553 PMC2630198

[41] Graci JD, Harki DA, Korneeva VS, Edathil JP, Too K, et al (2007) Lethal mutagenesis of poliovirus mediated by a mutagenic pyrimidine analogue. J Virol 81: 11256–11266. 17686844 10.1128/JVI.01028-07 PMC2045539

[42] Grande-Pérez A, Lazaro E, Lowenstein P, Domingo E, Manrubia SC (2005) Suppression of viral infectivity through lethal defection. Proc Natl Acad Sci USA 102: 4448–4452. 15767582 10.1073/pnas.0408871102 PMC555496

[43] Holland JJ (1990) Defective viral genomes. In: Fields BM, Knipe DM, editors. Virology. New York: Raven Press. 151–165.

[44] Lee CH, Gilbertson DL, Novella IS, Huerta R, Domingo E, et al (1997) Negative effects of chemical mutagenesis on the adaptive behavior of vesicular stomatitis virus. J Virol 71: 3636–3640. 9094637 10.1128/jvi.71.5.3636-3640.1997 PMC191512

[45] Loeb LA, Essigmann JM, Kazazi F, Zhang J, Rose KD, et al (1999) Lethal mutagenesis of HIV with mutagenic nucleoside analogs. Proc Natl Acad Sci USA 96: 1492–1497. 9990051 10.1073/pnas.96.4.1492 PMC15492

[46] Mullins JI, Heath L, Hughes JP, Kicha J, Styrchak S, et al (2011) Mutation of HIV-1 genomes in a clinical population treated with the mutagenic nucleoside KP1461. PLoS One 6: e15135. 21264288 10.1371/journal.pone.0015135 PMC3021505

[47] Pariente N, Sierra S, Lowenstein PR, Domingo E (2001) Efficient virus extinction by combinations of a mutagen and antiviral inhibitors. J Virol 75: 9723–9730. 11559805 10.1128/JVI.75.20.9723-9730.2001 PMC114544

[48] Perales C, Agudo R, Domingo E (2009) Counteracting quasispecies adaptability: extinction of a ribavirin-resistant virus mutant by an alternative mutagenic treatment. PLoS One 4: e5554. 19436746 10.1371/journal.pone.0005554 PMC2677667

[49] Perales C, Agudo R, Tejero H, Manrubia SC, Domingo E (2009) Potential benefits of sequential inhibitor-mutagen treatments of RNA virus infections. PLoS Pathog 5: e1000658. 19911056 10.1371/journal.ppat.1000658 PMC2771356

[50] Ruiz-Jarabo CM, Ly C, Domingo E, de la Torre JC (2003) Lethal mutagenesis of the prototypic arenavirus lymphocytic choriomeningitis virus (LCMV). Virology 308: 37–47. 12706088 10.1016/s0042-6822(02)00046-6

[51] Severson WE, Schmaljohn CS, Javadian A, Jonsson CB (2003) Ribavirin causes error catastrophe during Hantaan virus replication. J Virol 77: 481–488. 12477853 10.1128/JVI.77.1.481-488.2003 PMC140609

[52] Sierra S, Dávila M, Lowenstein PR, Domingo E (2000) Response of foot-and-mouth disease virus to increased mutagenesis. Influence of viral load and fitness in loss of infectivity. J Virol 74: 8316–8323. 10954530 10.1128/jvi.74.18.8316-8323.2000 PMC116341

[53] Tapia N, Fernandez G, Parera M, Gomez-Mariano G, Clotet B, et al (2005) Combination of a mutagenic agent with a reverse transcriptase inhibitor results in systematic inhibition of HIV-1 infection. Virology 338: 1–8. 15939449 10.1016/j.virol.2005.05.008

[54] Dapp MJ, Patterson SE, Mansky LM (2013) Back to the future: revisiting HIV-1 lethal mutagenesis. Trends Microbiol 21: 56–62. 23195922 10.1016/j.tim.2012.10.006 PMC3565075

[55] Crotty S, Cameron C, Andino R (2002) Ribavirin's antiviral mechanism of action: lethal mutagenesis? J Mol Med 80: 86–95. 11907645 10.1007/s00109-001-0308-0

[56] Crotty S, Maag D, Arnold JJ, Zhong W, Lau JYN, et al (2000) The broad-spectrum antiviral ribonucleotide, ribavirin, is an RNA virus mutagen. Nature Medicine 6: 1375–1379. 10.1038/82191 11100123

[57] Agudo R, Ferrer-Orta C, Arias A, de la Higuera I, Perales C, et al (2010) A multi-step process of viral adaptation to a mutagenic nucleoside analogue by modulation of transition types leads to extinction-escape. PLoS Pathog 6: e1001072. 20865120 10.1371/journal.ppat.1001072 PMC2928812

[58] Contreras AM, Hiasa Y, He W, Terella A, Schmidt EV, et al (2002) Viral RNA mutations are region specific and increased by ribavirin in a full-length hepatitis C virus replication system. J Virol 76: 8505–8517. 12163570 10.1128/JVI.76.17.8505-8517.2002 PMC136407

[59] Graci JD, Cameron CE (2002) Quasispecies, error catastrophe, and the antiviral activity of ribavirin. Virology 298: 175–180. 12127780 10.1006/viro.2002.1487

[60] Lanford RE, Chavez D, Guerra B, Lau JY, Hong Z, et al (2001) Ribavirin induces error-prone replication of GB virus B in primary tamarin hepatocytes. J Virol 75: 8074–8081. 11483752 10.1128/JVI.75.17.8074-8081.2001 PMC115051

[61] Moreno H, Gallego I, Sevilla N, de la Torre JC, Domingo E, et al (2011) Ribavirin can be mutagenic for arenaviruses. J Virol 85: 7246–7255. 21561907 10.1128/JVI.00614-11 PMC3126590

[62] Sierra M, Airaksinen A, González-López C, Agudo R, Arias A, et al (2007) Foot-and-mouth disease virus mutant with decreased sensitivity to ribavirin: implications for error catastrophe. J Virol 81: 2012–2024. 17151116 10.1128/JVI.01606-06 PMC1797574

[63] Kim Y, Lee C (2013) Ribavirin efficiently suppresses porcine nidovirus replication. Virus Res 171: 44–53. 23108045 10.1016/j.virusres.2012.10.018 PMC7114464

[64] Leyssen P, Balzarini J, De Clercq E, Neyts J (2005) The predominant mechanism by which ribavirin exerts its antiviral activity in vitro against flaviviruses and paramyxoviruses is mediated by inhibition of IMP dehydrogenase. J Virol 79: 1943–1947. 15650220 10.1128/JVI.79.3.1943-1947.2005 PMC544097

[65] Leyssen P, De Clercq E, Neyts J (2006) The anti-yellow fever virus activity of ribavirin is independent of error-prone replication. Mol Pharmacol 69: 1461–1467. 16421290 10.1124/mol.105.020057

[66] Asahina Y, Izumi N, Enomoto N, Uchihara M, Kurosaki M, et al (2005) Mutagenic effects of ribavirin and response to interferon/ribavirin combination therapy in chronic hepatitis C. J Hepatol. 43: 623–629. 10.1016/j.jhep.2005.05.032 16098627

[67] Chevaliez S, Brillet R, Lazaro E, Hezode C, Pawlotsky JM (2007) Analysis of ribavirin mutagenicity in human hepatitis C virus infection. J Virol 81: 7732–7741. 17494069 10.1128/JVI.00382-07 PMC1933365

[68] Lutchman G, Danehower S, Song BC, Liang TJ, Hoofnagle JH, et al (2007) Mutation rate of the hepatitis C virus NS5B in patients undergoing treatment with ribavirin monotherapy. Gastroenterology 132: 1757–1766. 17484873 10.1053/j.gastro.2007.03.035

[69] Gerotto M, Sullivan DG, Polyak SJ, Chemello L, Cavalletto L, et al (1999) Effect of retreatment with interferon alone or interferon plus ribavirin on hepatitis C virus quasispecies diversification in nonresponder patients with chronic hepatitis C. J Virol. 73: 7241–7247. 10.1128/jvi.73.9.7241-7247.1999 PMC104248 10438811

[70] Querenghi F, Yu Q, Billaud G, Maertens G, Trepo C, et al (2001) Evolution of hepatitis C virus genome in chronically infected patients receiving ribavirin monotherapy. J Viral Hepat 8: 120–131. 11264732 10.1046/j.1365-2893.2001.00265.x

[71] Sookoian S, Castano G, Frider B, Cello J, Campos R, et al (2001) Combined therapy with interferon and ribavirin in chronic hepatitis C does not affect serum quasispecies diversity. Dig Dis Sci 46: 1067–1071. 11341650 10.1023/a:1010718213584

[72] Perelson AS, Layden TJ (2007) Ribavirin: is it a mutagen for hepatitis C virus? Gastroenterology 132: 2050–2052. 17484896 10.1053/j.gastro.2007.03.077

[73] Zhou S, Liu R, Baroudy BM, Malcolm BA, Reyes GR (2003) The effect of ribavirin and IMPDH inhibitors on hepatitis C virus subgenomic replicon RNA. Virology 310: 333–342. 12781720 10.1016/s0042-6822(03)00152-1

[74] Cuevas JM, Gonzalez-Candelas F, Moya A, Sanjuan R (2009) Effect of ribavirin on the mutation rate and spectrum of hepatitis C virus in vivo. J Virol 83: 5760–5764. 19321623 10.1128/JVI.00201-09 PMC2681971

[75] Dixit NM, Layden-Almer JE, Layden TJ, Perelson AS (2004) Modelling how ribavirin improves interferon response rates in hepatitis C virus infection. Nature 432: 922–924. 15602565 10.1038/nature03153

[76] Kanda T, Yokosuka O, Imazeki F, Tanaka M, Shino Y, et al (2004) Inhibition of subgenomic hepatitis C virus RNA in Huh-7 cells: ribavirin induces mutagenesis in HCV RNA. J Viral Hepat 11: 479–487. 15500548 10.1111/j.1365-2893.2004.00531.x

[77] Dietz J, Schelhorn SE, Fitting D, Mihm U, Susser S, et al (2013) Deep sequencing reveals mutagenic effects of ribavirin during monotherapy of HCV genotype 1-infected patients. J Virol 87: 6172–6181. 23536652 10.1128/JVI.02778-12 PMC3648094

[78] Hultgren C, Milich DR, Weiland O, Sallberg M (1998) The antiviral compound ribavirin modulates the T helper (Th) 1/Th2 subset balance in hepatitis B and C virus-specific immune responses. J Gen Virol 79 (Pt 10): 2381–2391. 10.1099/0022-1317-79-10-2381 9780043

[79] Ning Q, Brown D, Parodo J, Cattral M, Gorczynski R, et al (1998) Ribavirin inhibits viral-induced macrophage production of TNF, IL-1, the procoagulant fgl2 prothrombinase and preserves Th1 cytokine production but inhibits Th2 cytokine response. J Immunol 160: 3487–3493. 9531310

[80] Feld JJ, Nanda S, Huang Y, Chen W, Cam M, et al (2007) Hepatic gene expression during treatment with peginterferon and ribavirin: Identifying molecular pathways for treatment response. Hepatology 46: 1548–1563. 17929300 10.1002/hep.21853 PMC2808168

[81] Zhang Y, Jamaluddin M, Wang S, Tian B, Garofalo RP, et al (2003) Ribavirin treatment up-regulates antiviral gene expression via the interferon-stimulated response element in respiratory syncytial virus-infected epithelial cells. J Virol 77: 5933–5947. 12719586 10.1128/JVI.77.10.5933-5947.2003 PMC154027

[82] Eriksson B, Helgstrand E, Johansson NG, Larsson A, Misiorny A, et al (1977) Inhibition of influenza virus ribonucleic acid polymerase by ribavirin triphosphate. Antimicrob Agents Chemother 11: 946–951. 879760 10.1128/aac.11.6.946 PMC352108

[83] Fernandez-Larsson R, O'Connell K, Koumans E, Patterson JL (1989) Molecular analysis of the inhibitory effect of phosphorylated ribavirin on the vesicular stomatitis virus in vitro polymerase reaction. Antimicrob Agents Chemother 33: 1668–1673. 2556073 10.1128/aac.33.10.1668 PMC172735

[84] Toltzis P, O'Connell K, Patterson JL (1988) Effect of phosphorylated ribavirin on vesicular stomatitis virus transcription. Antimicrob Agents Chemother 32: 492–497. 2837139 10.1128/aac.32.4.492 PMC172208

[85] Wray SK, Gilbert BE, Knight V (1985) Effect of ribavirin triphosphate on primer generation and elongation during influenza virus transcription in vitro. Antiviral Res 5: 39–48. 3985607 10.1016/0166-3542(85)90013-0

[86] Maag D, Castro C, Hong Z, Cameron CE (2001) Hepatitis C virus RNA-dependent RNA polymerase (NS5B) as a mediator of the antiviral activity of ribavirin. J Biol Chem 276: 46094–46098. 11602568 10.1074/jbc.C100349200

[87] Bougie I, Bisaillon M (2003) Initial binding of the broad spectrum antiviral nucleoside ribavirin to the hepatitis C virus RNA polymerase. J Biol Chem 278: 52471–52478. 14563844 10.1074/jbc.M308917200

[88] Streeter DG, Witkowski JT, Khare GP, Sidwell RW, Bauer RJ, et al (1973) Mechanism of action of 1- -D-ribofuranosyl-1,2,4-triazole-3-carboxamide (Virazole), a new broad-spectrum antiviral agent. Proc Natl Acad Sci USA 70: 1174–1178. 4197928 10.1073/pnas.70.4.1174 PMC433451

[89] Goswami BB, Borek E, Sharma OK, Fujitaki J, Smith RA (1979) The broad spectrum antiviral agent ribavirin inhibits capping of mRNA. Biochem Biophys Res Commun 89: 830–836. 226095 10.1016/0006-291x(79)91853-9

[90] Hofmann WP, Polta A, Herrmann E, Mihm U, Kronenberger B, et al (2007) Mutagenic effect of ribavirin on hepatitis C nonstructural 5B quasispecies in vitro and during antiviral therapy. Gastroenterology 132: 921–930. 17383421 10.1053/j.gastro.2006.12.005

[91] Brochot E, Duverlie G, Castelain S, Morel V, Wychowski C, et al (2007) Effect of ribavirin on the hepatitis C virus (JFH-1) and its correlation with interferon sensitivity. Antivir Ther 12: 805–813. 17713164

[92] Pfeiffer JK, Kirkegaard K (2005) Ribavirin resistance in hepatitis C virus replicon-containing cell lines conferred by changes in the cell line or mutations in the replicon RNA. J Virol 79: 2346–2355. 15681435 10.1128/JVI.79.4.2346-2355.2005 PMC546591

[93] Mori K, Ikeda M, Ariumi Y, Dansako H, Wakita T, et al (2011) Mechanism of action of ribavirin in a novel hepatitis C virus replication cell system. Virus Res 157: 61–70. 21320556 10.1016/j.virusres.2011.02.005

[94] Kato T, Date T, Miyamoto M, Sugiyama M, Tanaka Y, et al (2005) Detection of anti-hepatitis C virus effects of interferon and ribavirin by a sensitive replicon system. J Clin Microbiol 43: 5679–5684. 16272504 10.1128/JCM.43.11.5679-5684.2005 PMC1287837

[95] Iranzo J, Perales C, Domingo E, Manrubia SC (2011) Tempo and mode of inhibitor-mutagen antiviral therapies: A multidisciplinary approach. Proc Natl Acad Sci U S A 108: 16008–16013. 21911373 10.1073/pnas.1110489108 PMC3179121

[96] Perales C, Iranzo J, Manrubia SC, Domingo E (2012) The impact of quasispecies dynamics on the use of therapeutics. Trends Microbiol 20: 595–603. 22989762 10.1016/j.tim.2012.08.010

[97] Moreno H, Grande-Perez A, Domingo E, Martín V (2012) Arenaviruses and lethal mutagenesis. Prospects for new ribavirin-based interventions. Viruses 4: 2786–2805. 23202505 10.3390/v4112786 PMC3509673

[98] Lindenbach BD, Evans MJ, Syder AJ, Wolk B, Tellinghuisen TL, et al (2005) Complete replication of hepatitis C virus in cell culture. Science 309: 623–626. 15947137 10.1126/science.1114016

[99] Wakita T, Pietschmann T, Kato T, Date T, Miyamoto M, et al (2005) Production of infectious hepatitis C virus in tissue culture from a cloned viral genome. Nat Med 11: 791–796. 15951748 10.1038/nm1268 PMC2918402

[100] Zhong J, Gastaminza P, Cheng G, Kapadia S, Kato T, et al (2005) Robust hepatitis C virus infection in vitro. Proc Natl Acad Sci U S A 102: 9294–9299. 15939869 10.1073/pnas.0503596102 PMC1166622

[101] Marukian S, Jones CT, Andrus L, Evans MJ, Ritola KD, et al (2008) Cell culture-produced hepatitis C virus does not infect peripheral blood mononuclear cells. Hepatology 48: 1843–1850. 19003912 10.1002/hep.22550 PMC2592497

[102] Perales C, Henry M, Domingo E, Wain-Hobson S, Vartanian J (2011) P (2011) Lethal mutagenesis of foot-and-mouth disease virus involves shifts in sequence space. J Virol 85: 12227–12240. 21917974 10.1128/JVI.00716-11 PMC3209394

[103] Feng DF, Doolittle RF (1996) Progressive alignment of amino acid sequences and construction of phylogenetic trees from them. Methods in Enzymol 266: 368–382. 8743694 10.1016/s0076-6879(96)66023-6

[104] Zimmerman TP, Deeprose RD (1978) Metabolism of 5-amino-1-beta-D-ribofuranosylimidazole-4-carboxamide and related five-membered heterocycles to 5′-triphosphates in human blood and L5178Y cells. Biochem Pharmacol 27: 709–716. 207278 10.1016/0006-2952(78)90508-7

[105] Franklin TJ, Cook JM (1969) The inhibition of nucleic acid synthesis by mycophenolic acid. Biochem J 113: 515–524. 5807210 10.1042/bj1130515 PMC1184694

[106] Snell NJ (2001) Ribavirin-current status of a broad spectrum antiviral agent. Expert Opin Pharmacother 2: 1317–1324. 11585000 10.1517/14656566.2.8.1317

[107] Kerr SJ (1987) Ribavirin induced differentiation of murine erythroleukemia cells. Mol Cell Biochem 77: 187–194. 3481433 10.1007/BF00221928

[108] Lee HJ, Pawlak K, Nguyen BT, Robins RK, Sadee W (1985) Biochemical differences among four inosinate dehydrogenase inhibitors, mycophenolic acid, ribavirin, tiazofurin, and selenazofurin, studied in mouse lymphoma cell culture. Cancer Res 45: 5512–5520. 2865005

[109] Lowe JK, Brox L, Henderson JF (1977) Consequences of inhibition of guanine nucleotide synthesis by mycophenolic acid and virazole. Cancer Res 37: 736–743. 837373

[110] Sintchak MD, Fleming MA, Futer O, Raybuck SA, Chambers SP, et al (1996) Structure and mechanism of inosine monophosphate dehydrogenase in complex with the immunosuppressant mycophenolic acid. Cell 85: 921–930. 8681386 10.1016/s0092-8674(00)81275-1

[111] Henry SD, Metselaar HJ, Lonsdale RC, Kok A, Haagmans BL, et al (2006) Mycophenolic acid inhibits hepatitis C virus replication and acts in synergy with cyclosporin A and interferon-alpha. Gastroenterology 131: 1452–1462. 17101321 10.1053/j.gastro.2006.08.027

[112] Grande-Pérez A, Gómez-Mariano G, Lowenstein PR, Domingo E (2005) Mutagenesis-induced, large fitness variations with an invariant arenavirus consensus genomic nucleotide sequence. J Virol 79: 10451–10459. 16051837 10.1128/JVI.79.16.10451-10459.2005 PMC1182645

[113] González-López C, Gómez-Mariano G, Escarmís C, Domingo E (2005) Invariant aphthovirus consensus nucleotide sequence in the transition to error catastrophe. Infection Genetics and Evolution 5: 366–374. 10.1016/j.meegid.2005.05.001 16002345

[114] Domingo E, Sheldon J, Perales C (2012) Viral quasispecies evolution. Microbiology and Molecular Biology Reviews 76: 159–216. 22688811 10.1128/MMBR.05023-11 PMC3372249

[115] Moreno H, Tejero H, de la Torre JC, Domingo E, Martin V (2012) Mutagenesis-mediated virus extinction: virus-dependent effect of viral load on sensitivity to lethal defection. PLoS One 7: e32550. 22442668 10.1371/journal.pone.0032550 PMC3307711

[116] Baranovich T, Wong SS, Armstrong J, Marjuki H, Webby RJ, et al (2013) T-705 (Favipiravir) Induces Lethal Mutagenesis in Influenza A H1N1 Viruses In Vitro. J Virol 87: 3741–3751. 23325689 10.1128/JVI.02346-12 PMC3624194

[117] Dapp MJ, Clouser CL, Patterson S, Mansky LM (2009) 5-Azacytidine can induce lethal mutagenesis in human immunodeficiency virus type 1. J Virol 83: 11950–11958. 19726509 10.1128/JVI.01406-09 PMC2772699

[118] Solé RV, Deisboeck TS (2004) An error catastrophe in cancer? J Theor Biol 228: 47–54. 15064082 10.1016/j.jtbi.2003.08.018

[119] Loeb LA (2011) Human cancers express mutator phenotypes: origin, consequences and targeting. Nat Rev Cancer 11: 450–457. 21593786 10.1038/nrc3063 PMC4007007

[120] Fox EJ, Loeb LA (2010) Lethal mutagenesis: targeting the mutator phenotype in cancer. Semin Cancer Biol 20: 353–359. 20934515 10.1016/j.semcancer.2010.10.005 PMC3256989

[121] Parker WB (2005) Metabolism and antiviral activity of ribavirin. Virus Res 107: 165–171. 15649562 10.1016/j.virusres.2004.11.006

[122] Pan Q, de Ruiter PE, Metselaar HJ, Kwekkeboom J, de Jonge J, et al (2012) Mycophenolic acid augments interferon-stimulated gene expression and inhibits hepatitis C Virus infection in vitro and in vivo. Hepatology 55: 1673–1683. 22213147 10.1002/hep.25562

[123] Ye L, Li J, Zhang T, Wang X, Wang Y, et al (2012) Mycophenolate mofetil inhibits hepatitis C virus replication in human hepatic cells. Virus Res 168: 33–40. 22728816 10.1016/j.virusres.2012.06.009 PMC3505383

[124] Suzuki A, Miyawaki Y, Okuyama E, Murata M, Ando Y, et al (2013) Ribavirin-induced intracellular GTP depletion activates transcription elongation in coagulation factor VII gene expression. Biochem J 449: 231–239. 23050902 10.1042/BJ20121286

[125] Ando T, Imamura H, Suzuki R, Aizaki H, Watanabe T, et al (2012) Visualization and measurement of ATP levels in living cells replicating hepatitis C virus genome RNA. PLoS Pathog 8: e1002561. 22396648 10.1371/journal.ppat.1002561 PMC3291659

[126] Kim MG, Moon JS, Kim EJ, Lee SH, Oh JW (2012) Destabilization of PDK1 by Hsp90 inactivation suppresses hepatitis C virus replication through inhibition of PRK2-mediated viral RNA polymerase phosphorylation. Biochem Biophys Res Commun 421: 112–118. 22490666 10.1016/j.bbrc.2012.03.126

[127] Lehmann KA, Bass BL (2000) Double-stranded RNA adenosine deaminases ADAR1 and ADAR2 have overlapping specificities. Biochemistry 39: 12875–12884. 11041852 10.1021/bi001383g

[128] Nishikura K (2010) Functions and regulation of RNA editing by ADAR deaminases. Annu Rev Biochem 79: 321–349. 20192758 10.1146/annurev-biochem-060208-105251 PMC2953425

[129] Feld JJ, Lutchman GA, Heller T, Hara K, Pfeiffer JK, et al.. (2010) Ribavirin improves early responses to peginterferon through improved interferon signaling. Gastroenterology 139: 154–162 e154. 10.1053/j.gastro.2010.03.037 PMC2902566 20303352

[130] Rankin JT Jr, Eppes SB, Antczak JB, Joklik WK (1989) Studies on the mechanism of the antiviral activity of ribavirin against reovirus. Virology 168: 147–158. 2909988 10.1016/0042-6822(89)90413-3

[131] Gilbert BE, Knight V (1986) Biochemistry and clinical applications of ribavirin. Antimicrob Agents Chemother 30: 201–205. 2876677 10.1128/aac.30.2.201 PMC180518

[132] Connor E, Morrison S, Lane J, Oleske J, Sonke RL, et al (1993) Safety, tolerance, and pharmacokinetics of systemic ribavirin in children with human immunodeficiency virus infection. Antimicrob Agents Chemother 37: 532–539. 8460922 10.1128/aac.37.3.532 PMC187703

[133] Jordan I, Briese T, Fischer N, Lau JY, Lipkin WI (2000) Ribavirin inhibits West Nile virus replication and cytopathic effect in neural cells. J Infect Dis 182: 1214–1217. 10979920 10.1086/315847

[134] Crumpacker C, Bubley G, Lucey D, Hussey S, Connor J (1986) Ribavirin enters cerebrospinal fluid. Lancet 2: 45–46. 10.1016/s0140-6736(86)92590-0 2873344

[135] Ogle JW, Toltzis P, Parker WD, Alvarez N, McIntosh K, et al (1989) Oral ribavirin therapy for subacute sclerosing panencephalitis. J Infect Dis 159: 748–750. 2926165 10.1093/infdis/159.4.748

[136] Anderson JF, Rahal JJ (2002) Efficacy of interferon alpha-2b and ribavirin against West Nile virus in vitro. Emerg Infect Dis 8: 107–108. 11749765 10.3201/eid0801.010252 PMC2730275

[137] Slavenburg S, Huntjens-Fleuren HW, Dofferhoff TS, Richter C, Koopmans PP, et al (2011) Ribavirin plasma concentration measurements in patients with hepatitis C: early ribavirin concentrations predict steady-state concentrations. Ther Drug Monit 33: 40–44. 21191316 10.1097/FTD.0b013e318205f892

[138] Ojosnegros S, Agudo R, Sierra M, Briones C, Sierra S, et al (2008) Topology of evolving, mutagenized viral populations: quasispecies expansion, compression, and operation of negative selection. BMC Evol Biol 8: 207. 18637173 10.1186/1471-2148-8-207 PMC2515104

[139] Welker MW, Zeuzem S (2009) Occult hepatitis C: how convincing are the current data? Hepatology 49: 665–675. 19105211 10.1002/hep.22706

[140] Pham TN, Coffin CS, Michalak TI (2010) Occult hepatitis C virus infection: what does it mean? Liver Int 30: 502–511. 20070513 10.1111/j.1478-3231.2009.02193.x

[141] Maylin S, Martinot-Peignoux M, Moucari R, Boyer N, Ripault MP, et al (2008) Eradication of hepatitis C virus in patients successfully treated for chronic hepatitis C. Gastroenterology. 135: 821–829. 10.1053/j.gastro.2008.05.044 18593587

[142] Blight KJ, McKeating JA, Rice CM (2002) Highly permissive cell lines for subgenomic and genomic hepatitis C virus RNA replication. J Virol 76: 13001–13014. 12438626 10.1128/JVI.76.24.13001-13014.2002 PMC136668

[143] Jones CT, Catanese MT, Law LM, Khetani SR, Syder AJ, et al (2010) Real-time imaging of hepatitis C virus infection using a fluorescent cell-based reporter system. Nat Biotechnol 28: 167–171. 20118917 10.1038/nbt.1604 PMC2828266

[144] Vandamme A, Witvrouw M, Pannecouque C, Balzarini J, Van Laethem K, et al.. (2000) Evaluating Clinical Isolates for Their Phenotypic and Genotypic Resistance Against Anti-HIV Drugs; Kinchington D, Schinazi R, editors. Totowa, NJ: Humana Press Inc. 10.1385/1-59259-245-7:223 21331913

[145] Lindenbach BD, Rice CM (2003) Evasive maneuvers by hepatitis C virus. Hepatology 38: 769–771. 12939603 10.1002/hep.510380327

[146] Pogolotti AL Jr, Santi DV (1982) High-pressure liquid chromatography – ultraviolet analysis of intracellular nucleotides. Anal Biochem 126: 335–345. 7158770 10.1016/0003-2697(82)90524-3

[147] Sanchez-Jimenez C, Olivares I, de Avila Lucas AI, Toledano V, Gutierrez-Rivas M, et al (2012) Mutagen-mediated enhancement of HIV-1 replication in persistently infected cells. Virology 424: 147–153. 22265575 10.1016/j.virol.2011.12.016

[148] Sanchez G, Bosch A, Gomez-Mariano G, Domingo E, Pinto RM (2003) Evidence for quasispecies distributions in the human hepatitis A virus genome. Virology 315: 34–42. 14592757 10.1016/s0042-6822(03)00483-5

[149] Team RDC (2012) R: A language and environment for statistical computing. R foundation for Statistical Computing, Vienna, Austria.: ISBN 3-900051-900007-900050. Available: http://www.R-project.org/.

[150] Gentleman RC, Carey VJ, Bates DM, Bolstad B, Dettling M, et al (2004) Bioconductor: open software development for computational biology and bioinformatics. Genome Biol 5: R80. 15461798 10.1186/gb-2004-5-10-r80 PMC545600

[151] Pages H, Aboyoun P, Gentleman R, DebRoy S (2012) Biostrings: String objects representing biological sequences, and matching algorithms. R package version 2.24.1.

[152] Ramirez C, Gregori J, Buti M, Tabernero D, Camos S, et al (2013) A comparative study of ultra-deep pyrosequencing and cloning to quantitatively analyze the viral quasispecies using hepatitis B virus infection as a model. Antiviral Res 98: 273–283. 23523552 10.1016/j.antiviral.2013.03.007

[153] Huse SM, Huber JA, Morrison HG, Sogin ML, Welch DM (2007) Accuracy and quality of massively parallel DNA pyrosequencing. Genome Biol 8: R143. 17659080 10.1186/gb-2007-8-7-r143 PMC2323236

[154] Zagordi O, Klein R, Daumer M, Beerenwinkel N (2010) Error correction of next-generation sequencing data and reliable estimation of HIV quasispecies. Nucleic Acids Res 38: 7400–7409. 20671025 10.1093/nar/gkq655 PMC2995073

[155] Homs M, Buti M, Quer J, Jardi R, Schaper M, et al (2011) Ultra-deep pyrosequencing analysis of the hepatitis B virus preCore region and main catalytic motif of the viral polymerase in the same viral genome. Nucleic Acids Res 39: 8457–8471. 21742757 10.1093/nar/gkr451 PMC3201856

[156] Nei M, Li WH (1979) Mathematical model for studying genetic variation in terms of restriction endonucleases. Proc Natl Acad Sci U S A 76: 5269–5273. 291943 10.1073/pnas.76.10.5269 PMC413122

[157] Lindenbach BD (2009) Measuring HCV infectivity produced in cell culture and in vivo. Methods Mol Biol 510: 329–336. 19009272 10.1007/978-1-59745-394-3_24

