# Supplementary material for: Extinction of Hepatitis C Virus by Ribavirin in Hepatoma Cells Involves Lethal Mutagenesis
Source: PLoS One. 2013 Aug 16;8(8):e71039. doi: 10.1371/journal.pone.0071039 (PMC3745404; doi:10.1371/journal.pone.0071039)
Supplement: Table S1 — Mutations, corresponding amino acid and point accepted mutation (PAM) of the E2-coding region in the mutant spectra HCV p3 passaged in the absence or presence of ribavirin (Rib). (DOC) [file pone.0071039.s001.doc]

| **HCV p3 No drug** | | | **HCV p3 Rib 50 μM** | | | **HCV p3 Rib 100 μM** | | |
| --- | --- | --- | --- | --- | --- | --- | --- | --- |
| **Mutation^b^** | **Amino acid substitution^b^** | **PAM 250** | **Mutation^b^** | **Amino acid substitution^b^** | **PAM 250** | **Mutation^b^** | **Amino acid substitution^b^** | **PAM 250** |
| A1493T | T2S | 1 | C1532T | **R15C** | -4 | C1490T | **R1F** | -4 |
| A1583G | N32D | 2 | C1579G | **-** |  | C1491T | **R1F** | -4 |
| C1654T | - |  | A1583G | **N32D** | 2 | T1554C | **M22T** | -1 |
| T1658C | S57P | 1 | A1590G | **N34S** | 1 | C1636A | **-** |  |
| C1954T | - |  | C1624T | **-** |  | C1648T | **-** |  |
| A1960T | I157F | 1 | C1654T | **-** |  | T1651C | **-** |  |
| G1987A | - |  | A1670C | **T61P** | 0 | C1654T | **-** |  |
| T2020C | W177R | 2 | C1671T | **T61I** | 0 | A1670T | **T61S** | 1 |
| A2167G | K226E | 0 | G1987A | **-** |  | C1672T | **-** |  |
| T2203C | - |  | C2053T | **-** |  | T1679C | **F64L** | 2 |
| C2320T | - |  | G2193A | **R235K** | 3 | G1765A | **-** |  |
| T2341C | S284P | 1 | C2195T | **L236F** | 2 | G1892A | **V135M** | 2 |
| C2401T | - |  | C2212T | **-** |  | G1909A | **-** |  |
| T2568G | I360S | -1 | C2230T | **-** |  | G1963A | **-** |  |
|  |  |  | C2284T | **-** |  | T1972A | **-** |  |
|  |  |  | A2321C | **-** |  | G1987A | **-** |  |
|  |  |  | G2328A | **R280K** | 3 | A2054C | **R189S** | 0 |
|  |  |  | T2374C | **-** |  | G2112T | **R208M** | 0 |
|  |  |  | G2393A | **D302N** | 2 | C2135T | **L216F** | 2 |
|  |  |  | C2401T | **-** |  | C2143T | **-** |  |
|  |  |  | C2422A | **-** |  | C2146T | **-** |  |
|  |  |  | C2455T | **-** |  | G2307A | **C273Y** | 0 |
|  |  |  | G2533A | **-** |  | A2321C | **-** |  |
|  |  |  | C2550T | **A354V** | 0 | A2372G | **I295V** | 4 |
|  |  |  |  |  |  | T2374C | **-** |  |
|  |  |  |  |  |  | A2460G | **Y324C** | 0 |
|  |  |  |  |  |  | A2482G | **-** |  |
|  |  |  |  |  |  | G2531A | **A348T** | 1 |
|  |  |  |  |  |  | C2548T | **-** |  |
|  |  |  |  |  |  | C2569T | **-** |  |
|  |  |  |  |  |  | G2585A | **E366K** | 0 |
| **Total mutations^c^** | **14** |  | **Total mutations^c^** | **24** |  | **Total mutations^c^** | **31** |  |
| **Synonymous (%)^d^** | **6 (43)** |  | **Synonymous (%)^d^** | **14 (58)** |  | **Synonymous (%)^d^** | **17 (55)** |  |
| **Non-synonymous (%)^d^** | **8 (57)** |  | **Non-synonymous (%)^d^** | **10 (42)** |  | **Non-synonymous (%)^d^** | **14 (45)** |  |

**Table S1**. Mutations, corresponding amino acid and point accepted mutation (PAM) of the E2-coding region in the mutant spectra HCV p3 passaged in the absence or presence of ribavirin (Rib)^a^

^a^The populations are those described in Figures 3b, 4a and Table 1 of the main text.

^b^Mutation and deduced amino acid substitutions are relative to the sequence of the JFH-1 genome (accession number #AB047639). Amino acid residues (single letter code) are numbered from the N- to the C-terminus of E2. Boldface type indicates a change in the amino acid residue.

^c^Number of different mutations found comparing the sequence of each individual clone.

^d^Number of synonymous and non-synonymous mutations; their percentage is indicated in parenthesis.
