# Supplementary material for: Extinction of Hepatitis C Virus by Ribavirin in Hepatoma Cells Involves Lethal Mutagenesis
Source: PLoS One. 2013 Aug 16;8(8):e71039. doi: 10.1371/journal.pone.0071039 (PMC3745404; doi:10.1371/journal.pone.0071039)
Supplement: Table S2 — Mutations, corresponding amino acid and point accepted mutation (PAM) of the NS5A-coding region in the mutant spectra HCV p3 passaged in in the absence or presence of ribavirin (Rib). (DOC) [file pone.0071039.s002.doc]

**Table S2**. Mutations, corresponding amino acid and point accepted mutation (PAM) of the NS5A-coding region in the mutant spectra HCV p3 passaged in in the absence or presence of ribavirin (Rib)^a^

| **HCV p3 No drug** | | | **HCV p3 Rib 50 μM** | | | **HCV p3 Rib 100 μM** | | |
| --- | --- | --- | --- | --- | --- | --- | --- | --- |
| **Mutation^b^** | **Amino acid substitution^b^** | **PAM 250** | **Mutation^b^** | **Amino acid substitution^b^** | **PAM 250** | **Mutation^b^** | **Amino acid substitution^b^** | **PAM 250** |
| A6452G | **N62D** | 2 | C6298T | **-** |  | G6302A | **V12I** | 4 |
| A6455G | **I63V** | 4 | G6446T | **G60C** | -3 | C6339T | **T24I** | 0 |
| C6632T | **R122C** | -4 | A6452G | **N62D** | 2 | C6340T | **-** |  |
| G6641A | **G125R** | -3 | A6578G | **T104A** | 1 | C6355T | **-** |  |
| A6697G | **-** |  | C6768A | **P167Q** | 0 | A6452G | **N62D** | 2 |
| A6740G | **I158V** | 4 | C6955T | **-** |  | C6492T | **T75I** | 0 |
| A6758T | **T164S** | 1 | T7016C | **Y250H** | 0 | C6529A | **-** |  |
| G6784A | **-** |  | C7033A | **-** |  | T6535C | **-** |  |
| A6840G | **E191G** | 0 | A7035G | **D256G** | 1 | C6574T | **-** |  |
| A6877T | **-** |  | G7069T | **Q267H** | 3 | G6616A | **-** |  |
| T6980C | **S238P** | 1 | G7131C | **S288T** | 1 | G6631A | **-** |  |
| G7037A | **A257T** | 1 | A7158G | **E297G** | 0 | G6637A | **-** |  |
| C7057T | **-** |  | A7163G | **M299V** | 2 | C6721T | **-** |  |
| T7137A | **L290H** | -2 | C7169T | **P301S** | 1 | T6786C | **V173A** | 0 |
| T7137C | **L290P** | -3 | T7193C | **-** |  | G6815T | **A183S** | 1 |
| A7150G | **I294M** | 2 | G7210A | **-** |  | C6826T | **-** |  |
| A7163G | **M299V** | 2 | G7228T | **-** |  | C6853A | **D195E** | 3 |
| T7273C | **-** |  | T7273C | **-** |  | C6859T | **-** |  |
| T7338G | **V357G** | -1 | A7302C | **K345T** | 0 | G6902A | **E212N** | 1 |
| C7410T | **S381L** | -3 | G7304A | **A346T** | 1 | G6904T | **E212N** | 1 |
| A7533G | **E422G** | 0 | C7318T | **-** |  | G6922A | **-** |  |
| G7598A | **V444I** | 4 | T7371A | **L668H** | -2 | G6988A | **-** |  |
| A7655G | **T463A** | 1 | C7408T | **-** |  | T7016C | **Y250H** | 0 |
|  |  |  | T7409C | **S381P** | 1 | C7021T | **-** |  |
|  |  |  | G7415A | **G383S** | 1 | T7023C | **V252A** | 0 |
|  |  |  | G7439C | **A391P** | 1 | G7024T | **-** |  |
|  |  |  | C7447T | **-** |  | C7065A | **A266D** | 0 |
|  |  |  | G7460A | **G298S** | 1 | T7138G | **-** |  |
|  |  |  | T7501C | **-** |  | G7173A | **R302K** | 3 |
|  |  |  | A7533G | **E422G** | 0 | C7231T | **-** |  |
|  |  |  | T7572G | **L435R** | -3 | C7270T | **-** |  |
|  |  |  | C7585T | **-** |  | C7307T | **P347S** | 1 |
|  |  |  | G7598A | **V444I** | 4 | G7329C | **R354P** | 0 |
|  |  |  | G7612T | **-** |  | G7439C | **A391P** | 1 |
|  |  |  |  |  |  | C7447T | **-** |  |
|  |  |  |  |  |  | C7450T | **-** |  |
|  |  |  |  |  |  | G7457A | **G397S** | 1 |
|  |  |  |  |  |  | G7484A | **A406T** | 1 |
|  |  |  |  |  |  | C7487T | **P407S** | 1 |
|  |  |  |  |  |  | T7515C | **M416T** | -1 |
|  |  |  |  |  |  | A7533G | **E422G** | 0 |
|  |  |  |  |  |  | A7540T | **-** |  |
|  |  |  |  |  |  | C7574G | **Q436E** | 2 |
|  |  |  |  |  |  | G7588A | **-** |  |
| **Total mutations^c^** | **23** |  | **Total mutations^c^** | **34** |  | **Total mutations^c^** | **44** |  |
| **Synonymous (%)^d^** | **5 (22)** |  | **Synonymous (%)^d^** | **13 (38)** |  | **Synonymous (%)^d^** | **22 (50)** |  |
| **Non-synonymous (%)^d^** | **18 (78)** |  | **Non-synonymous (%)^d^** | **21 (62)** |  | **Non-synonymous (%)^d^** | **22 (50)** |  |

^a^The populations are those described in Figures 3b, 4a and Table 1 of the main text.

^b^Mutation and deduced amino acid substitutions are relative to the sequence of the JFH-1 genome (accession number #AB047639). Amino acid residues (single letter code) are numbered from the N- to the C-terminus of NS5A. Boldface type indicates a change in the amino acid residue.

^c^Number of different mutations found comparing the sequence of each individual clone.

^d^Number of synonymous and non-synonymous mutations; their percentage is indicated in parenthesis.
