# Supplementary material for: Extinction of Hepatitis C Virus by Ribavirin in Hepatoma Cells Involves Lethal Mutagenesis
Source: PLoS One. 2013 Aug 16;8(8):e71039. doi: 10.1371/journal.pone.0071039 (PMC3745404; doi:10.1371/journal.pone.0071039)
Supplement: Table S3 — Mutations, corresponding amino acid and point accepted mutation (PAM) of the NS5B-coding region in the mutant spectra HCV p3 passaged in the absence or presence of ribavirin (Rib). (DOC) [file pone.0071039.s003.doc]

**Table S3.** Mutations, corresponding amino acid and point accepted mutation (PAM) of the NS5B-coding region in the mutant spectra HCV p3 passaged in the absence or presence of ribavirin (Rib)

| **HCV p3 No drug** | | | **HCV p3 Rib 50 μM** | | | **HCV p3 Rib 100 μM** | | |
| --- | --- | --- | --- | --- | --- | --- | --- | --- |
| **Mutation^b^** | **Amino acid substitution^b^** | **PAM 250** | **Mutation^b^** | **Amino acid substitution^b^** | **PAM 250** | **Mutation^b^** | **Amino acid substitution^b^** | **PAM 250** |
| C7735T | **-** |  | T7741C | **-** |  | C7735T | **-** |  |
| C8191T | **-** |  | A7762C | **-** |  | C7798T | **-** |  |
| A8295G | **E210G** | 0 | A7786G | **-** |  | A7806T | **Q47L** | -2 |
| T8314G | **-** |  | A7840G | **-** |  | C7963T | **-** |  |
| G9144A | **G493D** | 1 | C7879T | **-** |  | G7970A | **G102R** | -3 |
| A9394T | **-** |  | T7919C | **-** |  | G8185A | **M173I** | 2 |
|  |  |  | A7965G | **K100R** | 3 | G8272A | **-** |  |
|  |  |  | C7993T | **-** |  | A8295G | **E210G** | 0 |
|  |  |  | C7996T | **-** |  | T8446G | **-** |  |
|  |  |  | C8001T | **S112F** | -3 | A8483G | **T273A** | 1 |
|  |  |  | C8029A | **-** |  | C8704T | **-** |  |
|  |  |  | C8132T | **P156S** | 1 | A8731G | **-** |  |
|  |  |  | G8164A | **-** |  | C8793T | **P376L** | -3 |
|  |  |  | G8185A | **M173I** | 2 | C8800T | **-** |  |
|  |  |  | C8191T | **-** |  | C8801T | **R379C** | -4 |
|  |  |  | C8210T | **L182F** | 2 | G8864A | **V400I** | 4 |
|  |  |  | G8221T | **-** |  | T8962C | **-** |  |
|  |  |  | A8295G | **E210G** | 0 | T8970A | **V435D** | -2 |
|  |  |  | C8347T | **-** |  | T9010C | **-** |  |
|  |  |  | C8583T | **A306V** | 0 | A9298G | **-** |  |
|  |  |  | C8662T | **-** |  | C9388T | **-** |  |
|  |  |  | G8668A | **-** |  |  |  |  |
|  |  |  | T8716G | **-** |  |  |  |  |
|  |  |  | C8792T | **P376S** | 1 |  |  |  |
|  |  |  | C8803T | **-** |  |  |  |  |
|  |  |  | C8939T | **-** |  |  |  |  |
|  |  |  | T9377G | **S571A** | 1 |  |  |  |
|  |  |  | C9392T | **-** |  |  |  |  |
| **Total mutations^c^** | **6** |  | **Total mutations^c^** | **28** |  | **Total mutations^c^** | **21** |  |
| **Synonymous (%)^d^** | **4 (67)** |  | **Synonymous (%)^d^** | **19 (73)** |  | **Synonymous (%)^d^** | **12 (57)** |  |
| **Non-synonymous (%)^d^** | **2 (33)** |  | **Non-synonymous (%)^d^** | **9 (27)** |  | **Non-synonymous (%)^d^** | **9 (43)** |  |

^a^The populations are those described in Figures 3b, 4a and Table 1 of the main text.

^b^Mutation and deduced amino acid substitutions are relative to the sequence of the JFH-1 genome (accession number #AB047639). Amino acid residues (single letter code) are numbered from the N- to the C-terminus of NS5B. Boldface type indicates a change in the amino acid residue.

^c^Number of different mutations found comparing the sequence of each individual clone.

^d^Number of synonymous and non-synonymous mutations; their percentage is indicated in parenthesis.
