# Supplementary material for: Extinction of Hepatitis C Virus by Ribavirin in Hepatoma Cells Involves Lethal Mutagenesis
Source: PLoS One. 2013 Aug 16;8(8):e71039. doi: 10.1371/journal.pone.0071039 (PMC3745404; doi:10.1371/journal.pone.0071039)
Supplement: Table S4 — Mutations, corresponding amino acid and point accepted mutation (PAM) of the NS5A-coding region in the mutant spectra HCV p4 and p5 passaged in the absence or presence of ribavirin (Rib). (DOC) [file pone.0071039.s004.doc]

**Table S4**. Mutations, corresponding amino acid and point accepted mutation (PAM) of the NS5A-coding region in the mutant spectra HCV p4 and p5 passaged in the absence or presence of ribavirin (Rib)^a^

| **HCV p4 No drug** | | | **HCV p5 No drug** | | | **HCV p4 R 75 μM** | | | **HCV p5 R 75 μM** | | | **HCV p4 R 100 μM** | | |
| --- | --- | --- | --- | --- | --- | --- | --- | --- | --- | --- | --- | --- | --- | --- |
| **Mutation^b^** | **Amino acid substitution^b^** | **PAM 250** | **Mutation^b^** | **Amino acid substitution^b^** | **PAM 250** | **Mutation^b^** | **Amino acid substitution^b^** | **PAM 250** | **Mutation^b^** | **Amino acid substitution^b^** | **PAM 250** | **Mutation^b^** | **Amino acid substitution^b^** | **PAM 250** |
| C6298T | **-** |  | C6335T | **-** |  | C6281T | **L5F** | 2 | T6278C | **W4R** | 2 | C6286T | **-** |  |
| G6568A | **-** |  | C6529G | **-** |  | C6528T | **T87I** | 0 | C6335T | **-** |  | C6412T | **-** |  |
| T6673C | **-** |  | T6733C | **-** |  | G6543T | **C92F** | -4 | C6364T | **-** |  | C6440T | **P58S** | 1 |
| T6679C | **-** |  | T6832C | **-** |  | A6636G | **Q123R** | 1 | C6595T | **-** |  | C6451T | **-** |  |
| G6693T | **C142F** | -4 | T7044C | **L259P** | -3 | C6676T | **-** |  | C6651T | **S128F** | -3 | C6463T | **-** |  |
| A6807G | **N180S** | 1 | T7145C | **S293P** | 1 | T6781C | **-** |  | G6739A | **-** |  | C6757T | **-** |  |
| T6832C | **-** |  | A7150G | **I294M** | 4 | C6859T | **-** |  | G6766C | **K166N** | 2 | A6763G | **-** |  |
| A7072G | **-** |  | A7207G | **-** |  | T6980C | **S238R** | 0 | C6775T | **-** |  | A7008G | **S247N** | 1 |
| A7134G | **D289G** | 1 | T7386C | **I373T** | 0 | G6989A | **A241T** | 1 | G6797T | **V177F** | -1 | A7011C | **N248T** | 0 |
| T7167A | **L300H** | -2 | G7438A | **-** |  | C6993T | **T242I** | 0 | A6808T | **K180N** | 1 | G7064A | **A266T** | 1 |
| T7409C | **S381P** | 1 | T7624C | **-** |  | C7003T | **-** |  | C6888T | **P207L** | -3 | G7087A | **-** |  |
| G7439C | **A391P** | 1 | A7655G | **T463A** | 1 | C7038G | **A257G** | 1 | G6913A | **-** |  | C7119T | **A284V** | 0 |
| T7462C | **-** |  |  |  |  | C7092T | **P275L** | -3 | C6939T | **P224L** | -3 | A7150G | **I294M** | 2 |
| A7540G | **-** |  |  |  |  | A7260G | **Q331R** | 1 | G6996A | **C243Y** | 0 | G7156A | **-** |  |
| A7655G | **T463A** | 1 |  |  |  | G7303T | **K345N** | 1 | C7042T | **-** |  | C7216T | **-** |  |
| G7658T | **V464L** | 2 |  |  |  | C7306T | **-** |  | T7047C | **L260P** | -3 | A7366G | **-** |  |
|  |  |  |  |  |  | G7364A | **E366K** | 0 | A7134G | **D289G** | 1 | C7370T | **L368F** | 2 |
|  |  |  |  |  |  | G7415A | **G383S** | 1 | T7279A | **-** |  | G7402A | **-** |  |
|  |  |  |  |  |  | G7441A | **-** |  | C7292T | **P342S** | 1 | C7449T | **A394V** | 0 |
|  |  |  |  |  |  | C7447T | **-** |  | T7371A | **L368H** | -2 | C7456T | **-** |  |
|  |  |  |  |  |  | C7517T | **P417S** | 1 | T7524C | **L419P** | -3 | C7481A | **P405T** | 0 |
|  |  |  |  |  |  | G7565C | **C433L** | -6 | G7564A | **-** |  | G7483T | **P405T** | 0 |
|  |  |  |  |  |  | C7632A | **T455N** | 0 | G7665A | **C466Y** | 0 | C7605T | **P446L** | -3 |
|  |  |  |  |  |  |  |  |  |  |  |  | G7618A | **-** |  |
|  |  |  |  |  |  |  |  |  |  |  |  | A7655G | **T463A** | 1 |
| **Total mutations^c^** | **16** |  | **Total mutations^c^** | **12** |  | **Total mutations^c^** | **23** |  | **Total mutations^c^** | **23** |  | **Total mutations^c^** | **25** |  |
| **Synonymous (%)^d^** | **8 (50)** |  | **Synonymous (%)^d^** | **7 (58)** |  | **Synonymous (%)^d^** | **7 (30)** |  | **Synonymous (%)^d^** | **9 (39)** |  | **Synonymous (%)^d^** | **13 (52)** |  |
| **Non-synonymous (%)^d^** | **8 (50)** |  | **Non-synonymous (%)^d^** | **5 (62)** |  | **Non-synonymous (%)^d^** | **16 (70)** |  | **Non-synonymous (%)^d^** | **14 (61)** |  | **Non-synonymous (%)^d^** | **12 (48)** |  |

^a^The populations are those described in Figure 3a, 4b and Table 2 of the main text.

^b^Mutation and deduced amino acid substitutions are relative to the sequence of the JFH-1 genome (accession number #AB047639). Amino acid residues (single letter code) are numbered from the N- to the C-terminus of NS5A. Boldface type indicates a change in the amino acid residue.

^c^Number of different mutations found comparing the sequence of each individual clone.

^d^Number of synonymous and non-synonymous mutations; their percentage is indicated in parenthesis.
