# Supplementary material for: Extinction of Hepatitis C Virus by Ribavirin in Hepatoma Cells Involves Lethal Mutagenesis
Source: PLoS One. 2013 Aug 16;8(8):e71039. doi: 10.1371/journal.pone.0071039 (PMC3745404; doi:10.1371/journal.pone.0071039)
Supplement: Table S5 — Mutations, corresponding amino acid and point accepted mutation (PAM) of the NS5A-coding region in the mutant spectra HCV p4 passaged in the absence or presence of ribavirin (Rib) analyzed by ultra deep sequencing. (DOC) [file pone.0071039.s005.doc]

**Table S5.** Mutations, corresponding amino acid and point accepted mutation (PAM) of the NS5A-coding region in the mutant spectra HCV p4 passaged in the absence or presence of ribavirin (Rib) analyzed by ultra deep sequencing^a^.

| **HCV p4 No drug** | | | | **HCV p4 Rib 75 μM** | | | |
| --- | --- | --- | --- | --- | --- | --- | --- |
| **Mutation^b^** | **Amino acid substitution^b^** | **PAM 250** | **Amplicon number** | **Mutation^b^** | **Amino acid substitution^b^** | **PAM 250** | **Amplicon number** |
| A6260G^c^ | **I259V** | 4 | 1 | C6159A^c^ | **T225K** | 0 | 1 |
| C6289U | **-** |  | 1 | G6172A^c^ | **-** |  | 1 |
| C6298U | **-** |  | 1 | G6184A^c^ | **-** |  | 1 |
| A6452G | **N62D** | 2 | 2 | C6187U^c^ | **-** |  | 1 |
| C6633A | **T122K** | 0 | 2 | C6203A^c^ | **L240I** | 2 | 1 |
| A6636G | **Q123R** | 1 | 2 | C6214U^c^ | **-** |  | 1 |
| U6716C | **F150L** | 1 | 2 | A6260G^c^ | **I259V** | 4 | 1 |
| U6733C | **-** |  | 2 | C6283U | **-** |  | 1 |
| U6741C | **I158T** | 0 | 2, 3 | A6319G | **-** |  | 1 |
| U6751C | **-** |  | 2, 3 | U6343A | **-** |  | 1 |
| C6757U | **-** |  | 3 | C6355U | **-** |  | 1 |
| U6781C | **-** |  | 3 | U6374G | **F36V** | -1 | 1 |
| U6816C | **-** |  | 3 | G6406C | **-** |  | 1 |
| A7020G | **D251G** | 1 | 4 | C6411U | **A48V** | 0 | 1 |
| A7026G | **D253G** | 1 | 4 | C6412U | **-** |  | 1 |
| A7070G | **T268A** | 1 | 4 | A6416G | **T50A** | 1 | 1 |
| G7139A | **E291K** | 0 | 4 | C6430U | **-** |  | 1 |
| A7148C | **I294L** | 2 | 4 | C6451U | **-** |  | 2 |
| A7150G | **I294M** | 2 | 4 | A6452G | **N62D** | 2 | 1, 2 |
| C7155U | **S296L** | -3 | 4 | C6473U | **-** |  | 2 |
| U7160C | **C298R** | -4 | 4 | C6490U | **-** |  | 2 |
| G7161A | **C298Y** | 0 | 4 | C6505U | **-** |  | 2 |
| A7163G | **M299V** | 2 | 4 | C6517A | **-** |  | 2 |
| U7164G | **M299T** | -1 | 4 | C6517U | **-** |  | 2 |
| U7164C | **M299R** | 0 | 4 | G6526A | **-** |  | 2 |
| U7183G | **F305L** | 1 | 4 | G6565U | **-** |  | 2 |
| U7201C | **-** |  | 4 | C6575U | **P103S** | 1 | 2 |
| G7228A | **-** |  | 4, 5 | C6576U | **P103L** | -3 | 2 |
| C7297A | **-** |  | 5 | C6577U | **-** |  | 2 |
| A7299C | **K344T** | 0 | 5 | A6581G | **N105D** | 2 | 2 |
| U7342C | **-** |  | 5 | C6592U | **-** |  | 2 |
| C7583U | **P439S** | 1 | 6 | C6595U | **-** |  | 2 |
| C7586A | **Q440K** | 1 | 6 | A6596G | **I110V** | 4 | 2 |
| A7655G | **T463A** | 1 | 6 | G6607A | **-** |  | 2 |
| G7658U | **V464L** | 2 | 6 | G6610A | **-** |  | 2 |
| U7661C | **C465R** | -4 | 6 | G6628A | **-** |  | 2 |
| U7661A | **C465S** | 0 | 6 | A6636G | **Q123R** | 1 | 2 |
| A7696G^d^ | **-** |  | 6 | U6640C | **-** |  | 2 |
|  |  |  |  | C6645U | **S126L** | -3 | 2 |
|  |  |  |  | C6676U | **-** |  | 2 |
|  |  |  |  | C6680U | **-** |  | 2 |
|  |  |  |  | U6733C | **-** |  | 2 |
|  |  |  |  | G6748A | **-** |  | 3 |
|  |  |  |  | G6779A | **D171N** | 2 | 3 |
|  |  |  |  | U6781C | **-** |  | 3 |
|  |  |  |  | G6784A | **-** |  | 3 |
|  |  |  |  | G6802A | **-** |  | 3 |
|  |  |  |  | C6803U | **L179F** | 1 | 3 |
|  |  |  |  | C6826U | **-** |  | 3 |
|  |  |  |  | C6843U | **P192L** | -3 | 3 |
|  |  |  |  | C6850U | **-** |  | 3 |
|  |  |  |  | G6854A | **A196T** | 1 | 3 |
|  |  |  |  | C6859U | **-** |  | 3 |
|  |  |  |  | C6889U | **-** |  | 3 |
|  |  |  |  | G6904A | **-** |  | 3 |
|  |  |  |  | G6910A | **-** |  | 3 |
|  |  |  |  | A6940G | **-** |  | 3 |
|  |  |  |  | G6913A | **-** |  | 4 |
|  |  |  |  | G6922A | **-** |  | 4 |
|  |  |  |  | C6948U | **A227V** | 0 | 4 |
|  |  |  |  | G6996A | **C243Y** | 0 | 4 |
|  |  |  |  | C7000U | **-** |  | 4 |
|  |  |  |  | C7003U | **-** |  | 4 |
|  |  |  |  | C7015U | **-** |  | 4 |
|  |  |  |  | A7020G | **D251G** | 1 | 4 |
|  |  |  |  | A7026U | **D253V** |  | 4 |
|  |  |  |  | G7034A | **D256N** | 2 | 4 |
|  |  |  |  | C7039U | **-** |  | 4 |
|  |  |  |  | U7047C | **L260P** | -3 | 4 |
|  |  |  |  | G7054A | **-** |  | 4 |
|  |  |  |  | C7057U | **-** |  | 4 |
|  |  |  |  | C7067U | **P270L** | -3 | 4 |
|  |  |  |  | A7080C | **E271A** | 0 | 4 |
|  |  |  |  | U7082C | **S272P** | 1 | 4 |
|  |  |  |  | A7101G | **D278G** | 1 | 4 |
|  |  |  |  | A7134G | **D289G** | 1 | 4 |
|  |  |  |  | G7139A | **E291K** | 0 | 4 |
|  |  |  |  | A7148C | **I294L** | 2 | 4 |
|  |  |  |  | A7150G | **I294M** | 2 | 4 |
|  |  |  |  | C7155U | **S296L** | -3 | 4 |
|  |  |  |  | G7161A | **C298Y** | 0 | 4 |
|  |  |  |  | A7163G | **M299V** | 2 | 4 |
|  |  |  |  | U7164G | **M299T** | -1 | 4 |
|  |  |  |  | U7164C | **M299R** | 0 | 4 |
|  |  |  |  | G7174U | **R302S** | 0 | 4 |
|  |  |  |  | G7180C | **-** |  | 4 |
|  |  |  |  | U7181C | **F305L** | 1 | 4 |
|  |  |  |  | U7183G | **F305L** | 1 | 4 |
|  |  |  |  | U7193C | **-** |  | 4 |
|  |  |  |  | G7198U | **-** |  | 4 |
|  |  |  |  | U7217C | **Y317H** | 0 | 4 |
|  |  |  |  | G7228A | **-** |  | 4, 5 |
|  |  |  |  | C7297A | **-** |  | 5 |
|  |  |  |  | A7299C | **K344T** | 0 | 5 |
|  |  |  |  | G7312A | **-** |  | 5 |
|  |  |  |  | U7342C | **-** |  | 5 |
|  |  |  |  | C7399U | **-** |  | 5 |
|  |  |  |  | C7403G | **P379A** | 1 | 5 |
|  |  |  |  | C7414U | **-** |  | 5 |
|  |  |  |  | G7436A | **G390R** | -3 | 6 |
|  |  |  |  | G7441A | **-** |  | 5, 6 |
|  |  |  |  | G7442A | **G392S** | 1 | 5 |
|  |  |  |  | C7447U | **-** |  | 5, 6 |
|  |  |  |  | C7450U | **-** |  | 6 |
|  |  |  |  | C7455U | **S396F** | -3 | 5 |
|  |  |  |  | C7456A | **-** |  | 5, 6 |
|  |  |  |  | G7457A | **G397S** | 1 | 5, 6 |
|  |  |  |  | C7459U | **-** |  | 6 |
|  |  |  |  | G7460A | **G398S** | 1 | 5, 6 |
|  |  |  |  | C7464A | **P399Q** | 0 | 5, 6 |
|  |  |  |  | C7464U | **P399L** | -3 | 5 |
|  |  |  |  | C7482U | **P405L** | -3 | 6 |
|  |  |  |  | G7484U | **A406S** | 1 | 5, 6 |
|  |  |  |  | C7497G | **T410R** | -1 | 5, 6 |
|  |  |  |  | C7510U | **-** |  | 6 |
|  |  |  |  | U7513C | **-** |  | 6 |
|  |  |  |  | G7516A | **M416I** | 2 | 5 |
|  |  |  |  | C7517U | **P417S** | 1 | 6 |
|  |  |  |  | C7518U | **P417L** | -3 | 5, 6 |
|  |  |  |  | C7525A | **-** |  | 5, 6 |
|  |  |  |  | U7556A | **S430T** | 1 | 6 |
|  |  |  |  | G7564A | **-** |  | 6 |
|  |  |  |  | C7605U | **P446L** | -3 | 6 |
|  |  |  |  | U7610C | **S448P** | 1 | 6 |
|  |  |  |  | G7621A | **-** |  | 6 |
|  |  |  |  | A7655G | **T463A** | 1 | 6 |
|  |  |  |  | A7696G^d^ | **-** |  | 6 |
| **Total mutations^e^** | **38** |  |  | **Total mutations^e^** | **127** |  |  |
| **Synonymous (%)^f^** | **12 (32)** |  |  | **Synonymous (%)^f^** | **68 (54)** |  |  |
| **Non-synonymous (%)^f^** | **26 (68)** |  |  | **Non-synonymous (%)^f^** | **59 (46)** |  |  |

^a^The populations are those described in Figure 3a, 4c and Table 3 of the main text.

^b^Mutation and deduced amino acid substitutions are relative to the sequence of the JFH-1 genome (accession number #AB047639). Amino acid residues (single letter code) are numbered from the N- to the C-terminus of NS5A. Boldface type indicates a change in the amino acid residue.

^c^Mutation and deduced amino acid substitutions correspond to NS4B-coding region.

^d^Mutation and deduced amino acid substitutions correspond to NS5B-coding region.

^e^Number of different mutations found comparing the sequence of each individual clone.

^f^Number of synonymous and non-synonymous mutations; their percentage is indicated in parenthesis.
