# Supplementary material for: Extinction of Hepatitis C Virus by Ribavirin in Hepatoma Cells Involves Lethal Mutagenesis
Source: PLoS One. 2013 Aug 16;8(8):e71039. doi: 10.1371/journal.pone.0071039 (PMC3745404; doi:10.1371/journal.pone.0071039)
Supplement: Table S6 — Mutations, corresponding amino acid and point accepted mutation (PAM) of the NS5A-coding region in the mutant spectra HCV p3 passaged in the absence or presence of ribavirin (Rib) and guanosine (Gua). (DOC) [file pone.0071039.s006.doc]

**Table S6.** Mutations, corresponding amino acid and point accepted mutation (PAM) of the NS5A-coding region in the mutant spectra HCV p3 passaged in the absence or presence of ribavirin (Rib) and guanosine (Gua)^a^

| **HCV p3 No drug** | | | **HCV p3 Gua 200 μM** | | | **HCV p3 R 50 μM + Gua 200 μM** | | | **HCV p3 R 100 μM + Gua 200 μM** | | |
| --- | --- | --- | --- | --- | --- | --- | --- | --- | --- | --- | --- |
| **Mutation^b^** | **Amino acid substitution^b^** | **PAM 250** | **Mutation^b^** | **Amino acid substitution^b^** | **PAM 250** | **Mutation^b^** | **Amino acid substitution^b^** | **PAM 250** | **Mutation^b^** | **Amino acid substitution^b^** | **PAM 250** |
| A6452G | **N62D** | 2 | A6357T | **K30I** | -2 | T6343C | **-** |  | T6269C | **S1L** | -3 |
| A6455G | **I63V** | 4 | G6358T | **K30I** | -2 | A6452G | **N62D** | 2 | C6270T | **S1L** | -3 |
| C6632T | **R122C** | -4 | G6400T | **K44N** | 1 | G6487A | **-** |  | T6347C | **-** |  |
| G6641A | **G125R** | -3 | G6420A | **G51D** | 1 | A6684G | **K139R** | 3 | A6452G | **N62D** | 2 |
| A6697G | **-** |  | A6452G | **N62D** | 2 | A6709G | **-** |  | G6511A | **M81I** | 2 |
| A6740G | **I158V** | 4 | T6474A | **L69Q** | -2 | A6758T | **T164S** | 1 | A6587G | **K107E** | 0 |
| A6758T | **T164S** | 1 | G6523A | **-** |  | C6762T | **P165L** | -3 | G6604A | **-** |  |
| G6784A | **-** |  | A6540C | **N91T** | 0 | T6812C | **Y182H** | 0 | G6608A | **A114T** | 1 |
| A6840G | **E191G** | 0 | G6634A | **-** |  | T6832C | **-** |  | G6610A | **-** |  |
| A6877T | **-** |  | G6641A | **G125R** | -3 | C6948T | **A227L** | -2 | G6628A | **-** |  |
| T6980C | **S238P** | 1 | A6683G | **K139E** | 0 | A6998G | **T244A** | 1 | C6718T | **-** |  |
| G7037A | **A257T** | 1 | A6696T | **Q143L** | -2 | A7013G | **T249A** | 1 | G6734A | **V156M** | 2 |
| C7057T | **-** |  | A6758T | **T164S** | 1 | A7068G | **Q267R** | 1 | C6757T | **-** |  |
| T7137A | **L290H** | -2 | A6940G | **-** |  | G7087A | **-** |  | A6758T | **T164S** | 1 |
| T7137C | **L290P** | -3 | A6998G | **T244A** | 1 | A7128G | **E287G** | 0 | C6820T | **-** |  |
| A7150G | **I294M** | 2 | C7033T | **-** |  | A7153G | **-** |  | A6828T | **Q187L** | -2 |
| A7163G | **M299V** | 2 | G7088A | **V274M** | 2 | T7167C | **L300P** | -3 | C6833T | **P189S** | 1 |
| T7273C | **-** |  | T7137C | **L290P** | -3 | T7201C | **-** |  | C6853T | **-** |  |
| T7338G | **V357G** | -1 | A7148G | **I294V** | 4 | A7252T | **-** |  | G6930A | **G221E** | 0 |
| C7410T | **S381L** | -3 | A7150G | **I294M** | 2 | A7268T | **T334S** | 1 | A6958C | **-** |  |
| A7533G | **E422G** | 0 | G7156T | **-** |  | C7294T | **-** |  | A6958T | **-** |  |
| G7598A | **V444I** | 4 | T7164C | **M299T** | -1 | G7300A | **-** |  | G6974A | **A236T** | 1 |
| A7655G | **T463A** | 1 | C7312T | **-** |  | A7302C | **K345T** | 0 | A7020C | **D251A** | 0 |
|  |  |  | A7391G | **D375G** | 1 | C7307T | **P347S** | 1 | T7023C | **V252A** | 0 |
|  |  |  | A7533G | **E422G** | 0 | C7318T | **-** |  | G7025A | **D253N** | 2 |
|  |  |  | G7598A | **V444I** | 4 | C7319T | **P351S** | 1 | G7034A | **D256N** | 2 |
|  |  |  |  |  |  | T7359C | **I364T** | 0 | A7035G | **D256G** | 1 |
|  |  |  |  |  |  | A7433G | **T389A** | 1 | G7063A | **-** |  |
|  |  |  |  |  |  | A7533G | **E422G** | 0 | T7089C | **V274A** | 0 |
|  |  |  |  |  |  | G7559A | **D431N** | 2 | T7103G | **F279V** | -1 |
|  |  |  |  |  |  | G7598A | **V444I** | 4 | A7115G | **M283V** | 2 |
|  |  |  |  |  |  | G7640C | **E458Q** | 2 | C7119T | **A284V** | 0 |
|  |  |  |  |  |  | A7655G | **T463A** | 1 | C7120T | **-** |  |
|  |  |  |  |  |  |  |  |  | C7132T | **-** |  |
|  |  |  |  |  |  |  |  |  | G7133A | **D289N** | 2 |
|  |  |  |  |  |  |  |  |  | A7134G | **D289G** | 1 |
|  |  |  |  |  |  |  |  |  | C7143T | **P292L** | -3 |
|  |  |  |  |  |  |  |  |  | A7150G | **I294M** | 2 |
|  |  |  |  |  |  |  |  |  | T7160C | **C298R** | -4 |
|  |  |  |  |  |  |  |  |  | G7180A | **-** |  |
|  |  |  |  |  |  |  |  |  | C7192T | **-** |  |
|  |  |  |  |  |  |  |  |  | C7224T | **P319L** | -3 |
|  |  |  |  |  |  |  |  |  | C7239T | **S324L** | -3 |
|  |  |  |  |  |  |  |  |  | G7246A | **-** |  |
|  |  |  |  |  |  |  |  |  | C7296T | **P343L** | -3 |
|  |  |  |  |  |  |  |  |  | T7344C | **L359P** | -3 |
|  |  |  |  |  |  |  |  |  | C7373A | **Q369K** | 1 |
|  |  |  |  |  |  |  |  |  | C7379T | **-** |  |
|  |  |  |  |  |  |  |  |  | G7382A | **A372T** | 1 |
|  |  |  |  |  |  |  |  |  | C7407T | **P380L** | -3 |
|  |  |  |  |  |  |  |  |  | G7438A | **-** |  |
|  |  |  |  |  |  |  |  |  | C7440T | **A391V** | 0 |
|  |  |  |  |  |  |  |  |  | G7461A | **G398D** | 1 |
|  |  |  |  |  |  |  |  |  | C7482T | **P405L** | -3 |
|  |  |  |  |  |  |  |  |  | C7486T | **-** |  |
|  |  |  |  |  |  |  |  |  | G7516C | **M416I** | 2 |
|  |  |  |  |  |  |  |  |  | C7521T | **P418L** | -3 |
|  |  |  |  |  |  |  |  |  | A7533G | **E422G** | 0 |
|  |  |  |  |  |  |  |  |  | C7544T | **P426S** | 1 |
|  |  |  |  |  |  |  |  |  | C7557A | **S430Y** | -3 |
|  |  |  |  |  |  |  |  |  | C7574G | **Q436E** | 2 |
|  |  |  |  |  |  |  |  |  | C7582T | **-** |  |
|  |  |  |  |  |  |  |  |  | G7598A | **V444I** | 4 |
|  |  |  |  |  |  |  |  |  | T7610C | **S448P** | 1 |
|  |  |  |  |  |  |  |  |  | G7612A | **-** |  |
|  |  |  |  |  |  |  |  |  | T7624G | **-** |  |
|  |  |  |  |  |  |  |  |  | A7655G | **T463A** | 1 |
| **Total mutations^c^** | **23** |  | **Total mutations^c^** | **26** |  | **Total mutations^c^** | **33** |  | **Total mutations^c^** | **67** |  |
| **Synonymous (%)^d^** | **5 (22)** |  | **Synonymous (%)^d^** | **6 (23)** |  | **Synonymous (%)^d^** | **11 (33)** |  | **Synonymous (%)^d^** | **22 (33)** |  |
| **Non-synonymous (%)^d^** | **18 (78)** |  | **Non-synonymous (%)^d^** | **20 (77)** |  | **Non-synonymous (%)^d^** | **22 (67)** |  | **Non-synonymous (%)^d^** | **45 (67)** |  |

^a^The populations are those described in Figure 5, 4d and Table 4 of the main text.

^b^Mutation and deduced amino acid substitutions are relative to the sequence of the JFH-1 genome (accession number #AB047639). Amino acid residues (single letter code) are numbered from the N- to the C-terminus of NS5A. Boldface type indicates a change in the amino acid residue.

^c^Number of different mutations found comparing the sequence of each individual clone.

^d^Number of synonymous and non-synonymous mutations; their percentage is indicated in parenthesis.
