# Supplementary material for: Extinction of Hepatitis C Virus by Ribavirin in Hepatoma Cells Involves Lethal Mutagenesis
Source: PLoS One. 2013 Aug 16;8(8):e71039. doi: 10.1371/journal.pone.0071039 (PMC3745404; doi:10.1371/journal.pone.0071039)
Supplement: Table S7 — Mutations, corresponding amino acid and point accepted mutation (PAM) of the NS5A-coding region in the mutant spectra HCV p3 passaged in the absence or presence of guanosine (Gua) and/or mycophenolic acid (MPA). (DOC) [file pone.0071039.s007.doc]

**Table S7.** Mutations, corresponding amino acid and point accepted mutation (PAM) of the NS5A-coding region in the mutant spectra HCV p3 passaged in the absence or presence of guanosine (Gua) and/or mycophenolic acid (MPA)^a^.

| **HCV p3 No drug** | | | **HCV p3 R Gua 200 μM** | | | **HCV p2 MPA 5 μM** | | | **HCV p3 MPA 5 μM + Gua 200 μM** | | |
| --- | --- | --- | --- | --- | --- | --- | --- | --- | --- | --- | --- |
| **Mutation^b^** | **Amino acid substitution^b^** | **PAM 250** | **Mutation^b^** | **Amino acid substitution^b^** | **PAM 250** | **Mutation^b^** | **Amino acid substitution^b^** | **PAM 250** | **Mutation^b^** | **Amino acid substitution^b^** | **PAM 250** |
| A6452G | **N62D** | 2 | A6357T | **K30I** | -2 | C6283T | **-** |  | A6319G | **-** |  |
| A6455G | **I63V** | 4 | G6358T | **K30I** | -2 | C6335T | **-** |  | A6425G | **M53V** | 2 |
| C6632T | **R122C** | -4 | G6400T | **K44N** | 1 | T6350C | **F28L** | 2 | A6452G | **N62D** | 2 |
| G6641A | **G125R** | -3 | G6420A | **G51D** | 1 | G6361A | **-** |  | C6615T | **S116L** | -3 |
| A6697G | **-** |  | A6452G | **N62D** | 2 | C6364A | **-** |  | A6696T | **Q143L** | -2 |
| A6740G | **I158V** | 4 | T6474A | **L69Q** | -2 | A6452G | **N62D** | 2 | C6835T | **-** |  |
| A6758T | **T164S** | 1 | G6523A | **-** |  | C6492T | **T75I** | 0 | T6863G | **L199V** | 2 |
| G6784A | **-** |  | A6540C | **N91T** | 0 | G6604A | **-** |  | A6940G | **-** |  |
| A6840G | **E191G** | 0 | G6634A | **-** |  | T6644A | **S126T** | 1 | A7020T | **D251V** | -2 |
| A6877T | **-** |  | G6641A | **G125R** | -3 | A6828G | **Q187R** | 1 | A7035G | **D256G** | 1 |
| T6980C | **S238P** | 1 | A6683G | **K139E** | 0 | C6900T | **A211V** | 0 | A7040G | **N258D** | 2 |
| G7037A | **A257T** | 1 | A6696T | **Q143L** | -2 | G6961C | **-** |  | C7057T | **-** |  |
| C7057T | **-** |  | A6758T | **T164S** | 1 | C6999T | **T244I** | 0 | T7078C | **-** |  |
| T7137A | **L290H** | -2 | A6940G | **-** |  | A7134G | **E289G** | 0 | A7115G | **M283V** | 2 |
| T7137C | **L290P** | -3 | A6998G | **T244A** | 1 | T7160C | **C298D** | -5 | T7164C | **M299T** | -1 |
| A7150G | **I294M** | 2 | C7033T | **-** |  | G7161A | **C298D** | -5 | T7167C | **L300P** | -3 |
| A7163G | **M299V** | 2 | G7088A | **V274M** | 2 | A7172G | **R302G** | -3 | A7327G | **-** |  |
| T7273C | **-** |  | T7137C | **L290P** | -3 | C7251T | **P328L** | -3 | C7393T | **-** |  |
| T7338G | **V357G** | -1 | A7148G | **I294V** | 4 | T7255C | **-** |  | G7442A | **G392S** | 1 |
| C7410T | **S381L** | -3 | A7150G | **I294M** | 2 | A7389G | **K374R** | 3 | G7495A | **-** |  |
| A7533G | **E422G** | 0 | G7156T | **-** |  | G7465A | **-** |  | A7514G | **M416V** | 2 |
| G7598A | **V444I** | 4 | T7164C | **M299T** | -1 | G7547A | **D427N** | 2 | A7533G | **S422G** | 1 |
| A7655G | **T463A** | 1 | C7312T | **-** |  | G7598A | **V444I** | 4 | G7598A | **V444M** | 2 |
|  |  |  | A7391G | **D375G** | 1 | A7631C | **T455P** | 0 | A7600G | **V444M** | 2 |
|  |  |  | A7533G | **E422G** | 0 | G7645A | **-** |  | A7631G | **T455A** | 1 |
|  |  |  | G7598A | **V444I** | 4 | A7655G | **T463A** | 1 | A7652G | **T462A** | 1 |
| **Total mutations^c^** | **23** |  | **Total mutations^c^** | **26** |  | **Total mutations^c^** | **26** |  | **Total mutations^c^** | **26** |  |
| **Synonymous (%)^d^** | **5 (22)** |  | **Synonymous (%)^d^** | **6 (23)** |  | **Synonymous (%)^d^** | **9 (35)** |  | **Synonymous (%)^d^** | **8 (31)** |  |
| **Non-synonymous (%)^d^** | **18 (78)** |  | **Non-synonymous (%)^d^** | **20 (77)** |  | **Non-synonymous (%)^d^** | **17 (65)** |  | **Non-synonymous (%)^d^** | **18 (69)** |  |

^a^The populations are those described in Figure 6, 4d and Table 5.

^b^Mutation and deduced amino acid substitutions are relative to the sequence of the JFH-1 genome (accession number #AB047639). Amino acid residues (single letter code) are numbered from the N- to the C-terminus of NS5A. Boldface type indicates a change in the amino acid residue.

^c^Number of different mutations found comparing the sequence of each individual clone.

^d^Number of synonymous and non-synonymous mutations; their percentage is indicated in parenthesis.
