# Supplementary material for: Extinction of Hepatitis C Virus by Ribavirin in Hepatoma Cells Involves Lethal Mutagenesis
Source: PLoS One. 2013 Aug 16;8(8):e71039. doi: 10.1371/journal.pone.0071039 (PMC3745404; doi:10.1371/journal.pone.0071039)
Supplement: Table S8 — Oligonucleotides used to amplify and sequence the HCV genomes. (DOC) [file pone.0071039.s008.doc]

**Table S8**. Oligonucleotides used to amplify and sequence the HCV genomes.

| Primer Name | Sequence (5’-3’) | Position^a^ |
| --- | --- | --- |
| **Jc1- E1-F1** | TCGCAGCCCAAATGTTCATTG | 1197-1217 |
| **Jc1-E2-F1** | ACATGGGCCCCAGGCAGAAAATCC | 1551-1574 |
| **Jc1-E2-R1** | ACAGTACACTGGGCCACACAC | 1850-1870 |
| **Jc1-E2-R2** | TTGCATGCAGCCGTGAGCCTG | 2266-2286 |
| **Jc1-p7-F1** | TCTTAGCGGACGCCAGGGTTTGC | 2526-2548 |
| **Jc1-p7-R1** | AGACGCGTCATAAGCATAAGC | 2771-2791 |
| **Jc1-NS5A F1** | ACTCAGAAGACTCCACAATTGG | 6220-6241 |
| **Jc1-NS5A F2** | ACTACCTTCTCCAGAGTTTTTC | 6697-6718 |
| **Jc1-NS5A R1** | TGGGTGCAAACCTATGGATCTG | 6737-6758 |
| **Jc1-NS5A F3** | TTTCCACGGGCCTTACCGGCTTG | 7181-7203 |
| **Jc1-NS5B F1** | TGGTCTACTTGCTCCGAGGAGG | 7625-7646 |
| **Jc1-NS5A R3** | ACTCAAAGGGTTGATTGGCAAC | 7726-7747 |
| **Jc1-NS5B F2** | CAAGAAACCAGCTCGCCTCATC | 8125-8146 |
| **Jc1-NS5B R1** | TCTCGCAGACCCGGACGCCGAG | 8159-8180 |
| **Jc1-NS5B F4** | AACCTCAACTTTGAGATGTATG | 8990-9011 |
| **Jc1-NS5B R4** | AGTTAGCTATGGAGTGTACCTAG | 9454-9476 |
| **Jc1-NS5B R5** | ACCTGGTCATGGCCTCCGTG | 8682-8702 |
| **Jc1-NS5B F6** | TGCCATACACTCGCTGACTGAGAG | 8419-8442 |
| **Jc1-NS5B R3** | TCTCAATTATGGCTGGAAGGTCC | 9037-9059 |
| **NS5A-T1F^b^** | CGTATCGCCTCCCTCGCGCCATCAG**AAACCACGTCGCCCCTACTCAC** | 6130-6151 |
| **NS5A-T1R^b^** | CTATGCGCCTTGCCAGCCCGCTCAG**AGGCGGACATTGCCAGAGAT** | 6455-6474 |
| **NS5A-T2F^b^** | CGTATCGCCTCCCTCGCGCCATCAG**TGACCACGCGCTGCCCTTGC** | 6426-6445 |
| **NS5A-T2R^b^** | CTATGCGCCTTGCCAGCCCGCTCAG**AGACCTCATCCCGGAAAAACG** | 6768-6788 |
| **NS5A-T3F^b^** | CGTATCGCCTCCCTCGCGCCATCAG**TTCTCCTGGGTGGACGGTGTG** | 6716-6736 |
| **NS5A-T3R^b^** | CTATGCGCCTTGCCAGCCCGCTCAG**TGCTGATAGCTGGCTCACTGAG** | 6955-6976 |
| **NS5A-T4F^b^** | CGTATCGCCTCCCTCGCGCCATCAG**ACATCACGGCGGAGACTGC** | 6891-6909 |
| **NS5A-T4R^b^** | CTATGCGCCTTGCCAGCCCGCTCAG**AACGGTGGGCGGTTGGTAATC** | 7253-7273 |
| **NS5A-T5F^b^** | CGTATCGCCTCCCTCGCGCCATCAG**TGGGCACGGCCTGACTACAACC** | 7202-7223 |
| **NS5A-T5R^b^** | CTATGCGCCTTGCCAGCCCGCTCAG**AAGCTCTACCTGATCAGACTCCA** | 7551-7573 |
| **NS5A-T6F^b^** | CGTATCGCCTCCCTCGCGCCATCAG**AGCGGTGATGCAGGCTCGTC** | 7412-7431 |
| **NS5A-T6R^b^** | CTATGCGCCTTGCCAGCCCGCTCAG**ACTCAAAGGGTTGATTGGCAAC** | 7726-7747 |

^a^According to JFH-1 (accession number #AB047639)

^b^Oligonucleotides used to perform ultra deep sequence analysis. Nucleotides underlined indicate HCV specific sequence
